# Overall lifestyle changes in adulthood are associated with cancer incidence in the Norwegian Women and Cancer Study (NOWAC) – a prospective cohort study

**DOI:** 10.1186/s12889-023-15476-3

**Published:** 2023-04-03

**Authors:** Sairah L. F. Chen, Therese H. Nøst, Edoardo Botteri, Pietro Ferrari, Tonje Braaten, Torkjel M. Sandanger, Kristin B. Borch

**Affiliations:** 1 Department of Community Medicine, UiT The Arctic University of Norway, Hansine Hansens Veg 18, 9019 Tromsø, Norway; 2 Department of Research, Cancer Registry of Norway, Ullernchauseen 64, 0379 Oslo, Norway; 3 Section for Colorectal Cancer Screening, Cancer Registry of Norway, Ullernchauseen 64, 0379 Oslo, Norway; 4 Nutrition and Metabolism Branch, International Agency for Research On Cancer, World Health Organization, World Health Organization, 150 Cours Albert Thomas, 69372 CEDEX 08 Lyon, France

**Keywords:** Lifestyle, Cancer, Women, Change, Prevention, Composite index, Repeated measurements, Cohort

## Abstract

**Background:**

Cancer is a leading cause of premature death worldwide and incidence is expected to rise in the coming decades. Many cohort studies, measuring lifestyle factors at one time-point, have observed that overall healthy lifestyles were inversely related to cancer incidence. However, there is little knowledge on the impact of lifestyle modification within adulthood.

**Methods:**

Using the Norwegian Women and Cancer study, two repeated self-reported assessments of lifestyle behaviours were used to calculate healthy lifestyle index scores at each time-point (*N* = 66 233). The associations between change in healthy lifestyle index score and lifestyle-related cancer incidence, including alcohol-, tobacco-, obesity-, and reproductive-related, and site-specific breast and colorectal cancer incidence were estimated using Cox proportional hazard regression models. To assess nonlinearity in the dose–response relationships, restricted cubic spline models were used.

**Results:**

Independent of baseline lifestyle, positive lifestyle changes were inversely related to the incidence of overall lifestyle-related cancers, as well as alcohol-related, tobacco-related, obesity-related, and reproductive-related cancers, but not breast and colorectal site-specific cancers. An association between lifestyle worsening and cancer incidence compared to stable lifestyle was observed.

**Conclusions:**

This study provides evidence that overall lifestyle changes among cancer-free women between the ages of 41 and 76 impact the incidence of many cancer types. Regardless of baseline lifestyle, there was a negative dose–response relationship between magnitude of positive lifestyle change and the incidence of overall lifestyle-related cancers. We observed that underlying this trend was an especially clear association between lifestyle worsening and increased risk compared to stable lifestyle. For adult women, maintaining a stable healthy lifestyle and lifestyle improvement are important for preventing the occurrence of many cancer types.

**Supplementary Information:**

The online version contains supplementary material available at 10.1186/s12889-023-15476-3.

## Background

Cancer is a major public health concern. As a leading cause of premature death worldwide [1] and projected to surpass premature deaths caused by cardiovascular diseases, the cancer burden is and will be devastating. The International Agency for Research on Cancer (IARC) estimated 19.3 million new cancer cases in 2020. Due to population growth and aging, the predicted number of new cancer cases will increase by 47% from 2022 to 2040 [2]. An increase in the prevalence of unhealthy lifestyle behaviours will intensify this burden.

Based on evidence collected primarily in high-income countries, approximately 40% of cancer cases are preventable [3]. Studies focused on individual lifestyle factors from baseline assessments constitute much of the evidence linking key modifiable factors, including lack of physical activity levels, overweight and obesity, smoking, alcohol intake, and poor dietary habits, to increased cancer risk [4]. The assumption that lifestyle measured at one time point during adulthood will be maintained throughout time is pervasive and indeed pragmatic in epidemiology. Prevention strategies rightfully seek to shift populations towards healthy behaviours throughout the life course. However, the estimates of risk difference founding this public health engagement lacks an important dimension – the impact of lifestyle modification within adulthood of the individual.

For single risk factor changes during adulthood, smoking cessation is perhaps the most established lifestyle modification known to prevent especially lung and upper aerodigestive tract cancers [5, 6]. Weight gain is associated with higher risk of postmenopausal breast and endometrial cancer according to several studies [7–9], but the results are inconsistent with respect to other cancers and weight loss [7, 10, 11]. Improved and stable cardiorespiratory fitness is inversely associated with overall cancer incidence compared to reduced cardiorespiratory fitness [12]. In the prospective Norwegian Women and Cancer Study (NOWAC), increased physical activity over assessments collected 6 to 8 years apart was inversely associated with only colon cancer risk [13]. Alcohol cessation has been shown to be associated with lower risk of several cancers [14–16], yet studies investigating the impact of graded changes in alcohol intake are few and inconclusive [17, 18]. To our knowledge, there are no studies exploring the association between changes in dietary habits alone and cancer risk. However, a randomised study observed that smoking cessation combined with dietary intervention reduced the risk of lifestyle-related cancers among men at high risk for cancer [19]. Changing several lifestyle factors has only been investigated in one additional study, observing that Swedish women who maintained or improved their lifestyle were at lower risk for lifestyle-related cancer compared to those who had consistently poor lifestyle [17]. However, the study did not include diet, which is an important element of lifestyle as it relates to cancer risk [4].

More evidence is required to understand the impact of lifestyle changes, involving individual and combined factors, on cancer risk. In this study the association between changing several lifestyle factors combined during adulthood, as measured by the healthy lifestyle index (HLI) score, on lifestyle-related cancer incidence was investigated in a cohort of Norwegian women.

## Methods

### Study sample and data collection

The NOWAC study has been described in detail previously [20] and has been used to investigate a wide range of lifestyle factors and health outcomes. In brief, the NOWAC study is a nationwide, prospective cohort consisting of approximately 172 000 adult female participants. Women invited to participate in the NOWAC study were randomly sampled from the Norwegian Central Person Register between 1991 and 2007 in multiple sub-cohorts. Consenting participants completed a self-administered questionnaire at enrolment and were invited to complete a maximum of three follow-up self-administered questionnaires, where each questionnaire was distributed between 2 and 11 years apart. All questionnaires, including follow-up questionnaires, collected information on socio-demographic characteristics, reproductive and hormonal factors, self-reported health, physical activity level, height, weight, smoking habits, dietary habits, and family history of breast cancer. Questionnaires consisted of either 4 or 8 pages depending on the sub-cohort, with the 8-page questionnaire containing a detailed food frequency questionnaire (FFQ). The first completed 8-page questionnaire was used as the baseline measurement for the present study (Q1) (Additional File 1). The subsequent completed 8-page follow-up questionnaire was used as the follow-up measurement (Q2) (Additional File 2). Participants that did not complete at least two 8-page questionnaires were excluded. In this study, Q1 was administered from 1996 to 2004 and Q2 was administered from 2002 to 2014.

**Fig. 1:**
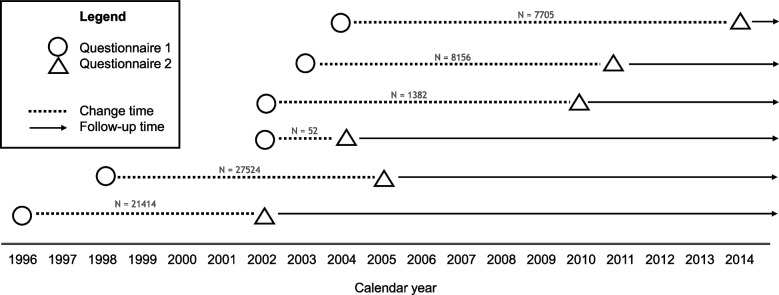
Timing of data collections and start of follow-up for the analytical sample, *N* = 66 233, Norwegian Women and Cancer Study (NOWAC)

The Norwegian personal identity number assigned to every resident of Norway and its linkage to the Cancer Registry of Norway, Cause of Death Register, and National Population register allowed for complete follow-up for all participants. Women who had died (*n* = 3), emigrated (*n* = 2) or had been diagnosed with cancer (*n* = 5018) before Q2 were excluded (see Additional File 3 for sample flow chart). A total of 66 233 participants were included in the analysis where 44 403 participants had complete information on lifestyle factors at two timepoints. The timeline of the final sample is shown in Fig. 1.

### Assessing lifestyle change

A healthy lifestyle index (HLI) was used to quantify overall lifestyle quality at Q1 and Q2. The construction of the HLI score in the NOWAC cohort was presented previously [21]. Briefly, the HLI used for this analysis consisted of five modifiable lifestyle factors – physical activity level, body fatness assessed by BMI (kg/m^2^), smoking behaviour, alcohol consumption (grams/day), and a dietary score. Physical activity level was reported by participants on a 1 to 10 scale ranging from not active to very active, where participants were asked to consider the entirety of activity at work, outside work, at home, exercise, and other forms of physical activity. Smoking behaviour was measured by smoking status, time since cessation for former smokers, and current number of cigarettes smoked per day. Each lifestyle factor was assigned a score ranging from 0 to 4, which were summed to a total HLI score that ranged from 0 to 20, where higher scores indicated a healthier lifestyle. See Additional File 4 for details on HLI construction. The HLI score change was the difference between HLI score at Q2 and Q1, where positive score changes represented lifestyle improvement and negative score changes represented lifestyle worsening.

### Outcome ascertainment

Follow-up time began at the end of Q2 and lasted until December 2018. Date of death and emigration were obtained through linkage to the Central Population Registry of Norway. Cancer diagnosis and date of diagnosis were obtained through linkage to the Cancer Registry of Norway based on codes from the International Classification of Diseases, Tenth Revision (ICD-10). The present study investigated all cancers considered to be lifestyle-related, constituting this study’s total cancer cases, and several cancer subgroupings including alcohol-related, tobacco-related, obesity-related, and reproductive-related cancers based on the IARC monograph on known causes and prevention by organ site (Additional File 5) [22]. Breast and colorectal cancer incidence were also investigated separately.

### Statistical analysis

Cox proportional hazards regression model, with age as the time scale, was used to estimate hazard ratios (HR) and 95% confidence intervals (CI). Age at entry was participants’ age at Q2 and age at exit was age at cancer diagnosis, death, emigration, or age in 31 December 2018, whichever occurred first. Associations were estimated between continuous (per 1 SD increase) and categorical change in HLI score and incidence of alcohol-related, tobacco-related, obesity-related, breast- and reproductive-related, and lifestyle-related cancer incidence. Seven categories for HLI score change were used: ≤ 3, 2, and 1 point decrease, stable, 1, 2, and ≥ 3 point increase. The proportional hazards assumption was tested using Schoenfeld residuals. Potential non-linear associations were tested with restricted cubic splines, modelled with three knots located at the predictor minimum and maximum, and the remaining at the equidistant percentile (50^th^), as recommended by Harrell [23]. Likelihood ratio tests were used to compare goodness of fit between non-linear and linear models.

The confounders included in the models were based on previous literature and determined a priori. They included education (years), height (centimetres), HLI score at Q1 (continuous) and calendar year at Q2 (continuous). Alcohol-related, obesity-related, reproductive-related, breast, and lifestyle-related cancer models were additionally adjusted for age at menarche (years), menopausal status (premenopausal/postmenopausal), breastfeeding (cumulative months 0, <  = 12, > 12 months), hormone replacement therapy use (current, former, never), oral contraceptive use (ever, never), parity (0, 1–2, > 2), and history of breast cancer in first degree relatives (yes, no). Perimenopausal women were considered premenopausal. Women missing on menopausal status were reported as postmenopausal if age 53 or older at the time of Q2.

The associations between individual index components, modelled as single lifestyle factor scores (continuous), and all outcomes were estimated using the same outcome-based adjustment sets described above. All single lifestyle factor scores were included in the model as they were the exposures of interest and mutually adjusted for one another. Correlation between HLI score changes across years between Q1 and Q2 was assessed with the Pearson correlation coefficient.

### Sensitivity analysis

To assess the contribution of each lifestyle factor to the associations between HLI score change (continuous) and cancer outcomes, the scores for physical activity, BMI, smoking, alcohol, and diet were excluded, one by one, from the HLI. Potential period effects were tested by performing analysis within two stratified enrolment year groups (1996–98, 2002–04) detailed in Fig. 1. The presence of effect modifications by age at Q1, age at Q2, and Q1 HLI score categories (HLI score 0–11, 12–13, 14–15, 16–20) were tested by modelling interaction terms and comparing models including the interaction term to the model without the interaction term using the likelihood ratio test. The first two years of follow-up time were excluded to test the impact of intentional or unintentional lifestyle changes due to morbid conditions, included pre-diagnosed cancer. The association between HLI change and all remaining cancers (not lifestyle-related) was estimated to test the viability of the lifestyle-related cancer grouping.

### Multiple imputation

Missing data among variables constituting the HLI score at Q1 and/or Q2, and covariates for 21 830 participants were handled by multiple imputation chained equations (MICE) under the assumption that data were missing at random [24]. All covariates included in the cancer models and the Nelson Aalen cumulative hazard estimator were included in the MICE model. MICE analysis employed fully conditional specification, whereby each incomplete variable was modelled iteratively by a series of multivariable regression models [25]. A total of 100 datasets were generated with 10 iterations each. Parameter estimates in the Cox models from each imputed dataset were averaged through the Rubin’s rule [26] to account for uncertainty in the MICE models to impute missing values. Model parameters were also estimated in complete-case analyses. Descriptive statistics for each imputed variable were compared between observed and imputed values. Convergence of MICE models were assessed by visual inspection of plots of the mean HLI score change against iteration number for each MI dataset (not shown).

All data treatment and statistical analysis were conducted in RStudio Version 1.2.959 with R Version 4.0.3 [27]. All statistical hypotheses were tested two-sided, allowing a Type I error rate of 5%.

## Results

At the start of follow-up (Q2), the mean age was 58.2 years, 46% of participants reported a physical activity level ≥ 6 on the NOWAC 1–10 scale, mean BMI was 25.4 (kg/m^2^), 20.7% were current smokers, median daily intake of alcohol was 2.09 g/day (mean: 4.0 g/day; IQR: 0.6, 5.8), the median expanded diet score was 9, and the median HLI score was 13 (Table 1). The mean time between Q1 and Q2 was 7 years (range: 2 – 11) with a mean HLI score change of -0.2 (range: -11 to 14). There was no correlation between the number of years between Q1 and Q2 and HLI score change (r = -0.06). The largest proportion of participants exhibited an HLI score difference of zero (17.2%), followed by decrease of three points (16.1%), decrease of one point (16.0%), increase of one point (15.2%), decrease of two points (12.7%), increase of three points (12.3%), and increase of two points (10.5%). The distributions of HLI change scores within Q1 HLI groups (Q1 HLI score group 0–11, 12–13, 14–15, 16–20) were different across Q1 HLI groups, reflecting the constraints of maximum and minimum change on the HLI and thus the probability distribution (Additional File 6).

**Table 1:** Characteristics of the study population at the start of follow-up (Questionnaire 2) according to healthy lifestyle index score change in the Norwegian Women and Cancer Study (*N* = 66,233)

		**HLI score change**							
	**Total (*N* = 66,233)**	**Decrease 3 or more (*N* = 7169)**	**Decrease 2 (*N* = 5636)**	**Decrease 1 (*N* = 7099)**	**Stable (*N* = 7638)**	**Increase 1 (*N* = 6743)**	**Increase 2 (*N* = 4673)**	**Increase 3 or more (*N* = 5445)**	**Missing, N (%)**
Age(years)	58.2 (6.3)	57.2 (5.9)	57.3 (6.0)	57.2 (5.9)	57.3 (6.0)	57.3 (6.1)	57.4 (6.2)	57.3 (6.0)	0(0)
Education (years)	12.3 (3.5)	12.5 (3.4)	12.7 (3.4)	12.6 (3.4)	12.7 (3.5)	12.6 (3.4)	12.6 (3.4)	12.5 (3.4)	3471 (5%)
HLI score at Q2, median (IQR)	136 (11, 15)	10 (9, 12)	12 (10, 14)	13 (10, 14)	13 (11, 15)	14 (12, 15)	14 (12, 16)	14 (13, 16)	16,343 (25%)
Physical activity score change	0.1 (1.4)	-1.2 (1.3)	-0.5 (1.1)	-0.2 (1.1)	0.1 (1.0)	0.5 (1.1)	0.8 (1.1)	1.5 (1.3)	11,969 (18%)
BMI score change	-0.3 (0.8)	-0.7 (0.8)	-0.5 (0.7)	-0.4 (0.7)	-0.2 (0.7)	-0.1 (0.7)	-0.0 (0.7)	0.2 (0.8)	3713 (6%)
Smoking score change	0.1 (0.5)	-0.0 (0.6)	0.0 (0.5)	0.1 (0.5)	0.1 (0.5)	0.1 (0.5)	0.2 (0.5)	0.3 (0.6)	5939 (9%)
Alcohol score change	-0.1 (0.7)	-0.4 (0.7)	-0.2 (0.7)	-0.2 (0.6)	-0.1 (0.6)	-0.0 (0.6)	0.0 (0.6)	0.2 (0.7)	2920 (4%)
Diet score change	0.0 (1.6)	-1.5 (1.4)	-0.8 (1.2)	-0.4 (1.2)	0.1 (1.1)	0.6 (1.2)	1.0 (1.2)	1.6 (1.3)	6914 (10%)
Height (cm)	166.0 (5.7)	166.2 (5.7)	166.4 (5.7)	166.1 (5.6)	166.3 (5.7)	166.2 (5.6)	166.3 (5.7)	166.4 (5.6)	1705 (3%)
Physical activity level (> = 6)^a^, N (%)	30,391 (46%)	2182 (30%)	2457 (44%)	2457 (44%)	4262 (56%)	4064 (60%)	3041 (65%)	3956 (73%)	8572 (13%)
Body mass index (kg/m^2^)	25.4 (4.2)	26.3 (4.1)	25.7 (4.1)	25.5 (4.1)	25.1 (4.1)	25.1 (4.2)	24.9 (4.1)	25.0 (4.1)	2633 (4%)
Smoking status, N (%)									2913 (4%)
Never	22,653 (34%)	2475 (35%)	2189 (39%)	2726 (38%)	3007 (39%)	2550 (38%)	1724 (37%)	1650 (30%)	
Former	26,942 (41%)	2986 (42%)	2263 (40%)	2907 (41%)	3090 (40%)	2751 (41%)	2012 (43%)	2687 (49%)	
Current	13,725 (21%)	1708 (24%)	1184 (21%)	1466 (21%)	1541 (20%)	1442 (21%)	937 (20%)	1108 (20%)	
Alcohol intake (g/day)	4.0 (5.0)	5.1 (5.8)	4.5 (5.2)	4.4 (5.3)	4.2 (4.9)	4.0 (4.9)	3.9 (4.5)	3.8 (4.6)	3387 (5%)
Diet score (0–18)	8.8 (2.5)	7.3 (2.3)	8.0 (2.4)	8.5 (2.5)	9.0 (2.5)	9.3 (2.3)	9.6 (2.2)	9.9 (2.1)	4786 (7%)
Postmenopausal, N (%)	52,110 (79%)	5415 (76%)	4270 (76%)	5346 (75%)	5798 (76%)	5048 (75%)	3528 (75%)	4111 (76%)	0(0)
Age at menarche (years)	13.3 (1.4)	13.2 (1.4)	13.3 (1.4)	13.3 (1.4)	13.3 (1.4)	13.3 (1.4)	13.3 (1.4)	13.3 (1.4)	921 (1%)
Hormone replacement therapy status, N (%)									0(0)
Never	45,276 (68%)	4970 (69%)	3865 (69%)	4924 (69%)	5260 (69%)	4521 (67%)	3158 (68%)	3640 (67%)	
Former	6852 (10%)	705 (10%)	546 (10%)	650 (9%)	737 (10%)	705 (10%)	479 (10%)	550 (10%)	
Current	14,105 (21%)	1494 (21%)	1225 (22%)	1525 (21%)	1641 (21%)	1517 (22%)	1036 (22%)	1255 (23%)	
Oral contraceptive ever use, N (%)	35,451 (54%)	4205 (59%)	3262 (58%)	3993 (56%)	4344 (57%)	3823 (57%)	2669 (57%)	3143 (58%)	0(0)
Parity, N (%)									0(0)
0	5411 (8%)	576 (8%)	475 (8%)	580 (8%)	611 (8%)	585 (9%)	398 (9%)	465 (9%)	
1–2	34,666 (52%)	3825 (53%)	2987 (53%)	3850 (54%)	4149 (54%)	3667 (54%)	2524 (54%)	3056 (56%)	
> 2	26,156 (39%)	2768 (39%)	2174 (39%)	2669 (38%)	2878 (38%)	2491 (37%)	1751 (37%)	1924 (35%)	
Cumulative breastfeeding duration (months), N (%)									0(0)
0	34,402 (52%)	3950 (55%)	3229 (57%)	4063 (57%)	4351 (57%)	3827 (57%)	2696 (58%)	3023 (56%)	
< = 12	16,797 (25%)	1622 (23%)	1251 (22%)	1585 (22%)	1716 (22%)	1599 (24%)	1116 (24%)	1350 (25%)	
> 12	15,034 (23%)	1597 (22%)	1156 (21%)	1451 (20%)	1571 (21%)	1317 (20%)	861 (18%)	1072 (20%)	
Family history of breast cancer in the first degree, N (%)	5180 (8%)	559 (8%)	421 (7%)	570 (8%)	571 (7%)	506 (8%)	366 (8%)	385 (7%)	(0)

Values are mean (SD) unless otherwise specified

^a^ Presents the physical activity level on the NOWAC 1–10 scale

The median follow-up time was 14.2 years during which 6 384 lifestyle-related cancer cases occurred, reflecting the total number of cancer cases. Within overlapping cancer groupings, there were 3 512 alcohol-related, 2 931 tobacco-related, 4 788 obesity-related, 3 385 reproductive-related, 2 384 breast, and 839 colorectal cancer cases that occurred.

The estimates obtained from MICE data models were within ± 5% of those obtained from complete-case data models for continuous exposure models and demonstrated a similar trend for categorical exposure models (see complete-case results in Additional File 7). Therefore, all presented estimates were obtained from MICE data models, except for those in figures displaying HLI score change modelled with restricted cubic splines, where estimates from complete-case data models were described.

**Fig. 2:**
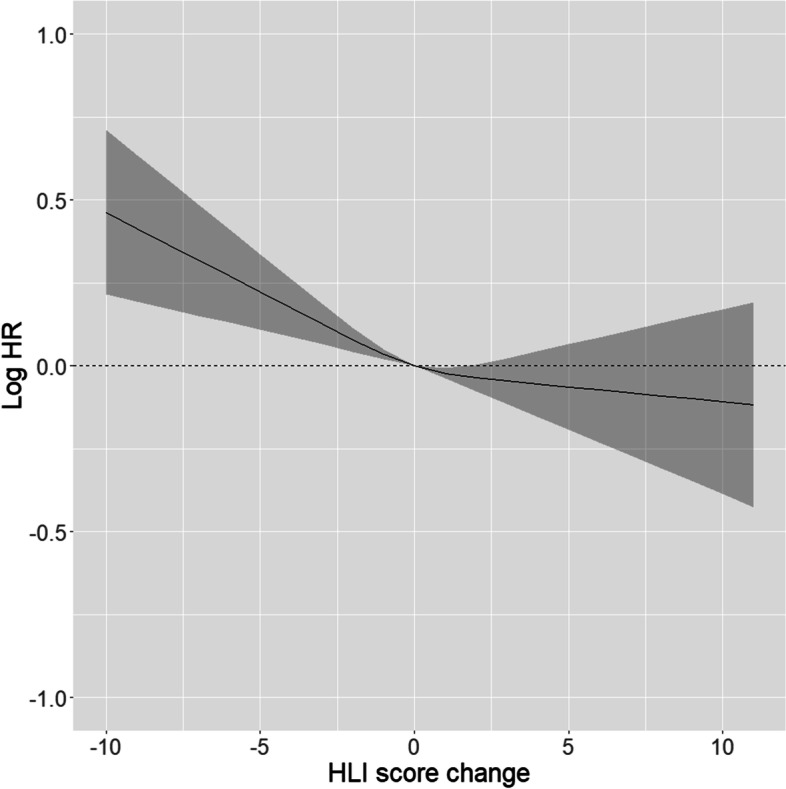
Association between HLI score change modelled using restricted cubic splines and lifestyle-related cancer incidence

After adjusting for covariates, for every 1 SD increase in HLI score change, the HR was 0.93 (95% CI: 0.90–0.96) for lifestyle-related cancers, 0.96 (95% CI: 0.91–0.99) for alcohol-related cancers, 0.92 (95% CI: 0.88–0.96) for tobacco-related cancers, 0.94 (95% CI: 0.91–0.98) for obesity-related cancers, 0.90 (95% CI: 0.84–0.98) for reproductive-related cancers, 0.96 (95% CI: 0.91–1.01) for breast cancer, and 0.98 (95% CI: 0.90–1.07) for colorectal cancer (Table 2). When the HLI score change was modelled using restricted cubic splines, there were no indications of nonlinearity (all p-values > 0.05) in the adjusted associations for all lifestyle-related cancers (Fig. 2) nor for all other outcomes (Additional File 8).

**Table 2:** Associations between healthy lifestyle index score change and lifestyle-related, alcohol-related, tobacco-related, obesity-related, reproductive-related, breast, and colorectal cancer incidence in the Norwegian Women and Cancer Study (*n* = 66,233), imputed analysis

		**Lifestyle-related cancer incidence**^**a**^	**Alcohol-related cancer incidence**^**a**^	**Tobacco-related cancer incidence**	**Obesity-related cancer incidence**^**a**^	**Reproductive-related cancer incidence**^**a**^	**Breast cancer incidence**^**a**^	**Colorectal cancer incidence**
**Cases**		6354	3512	2931	4788	3385	2384	839
**Continuous HLI score change**	1-SD (2.6 HLI points) increase	0.93(0.90–0.96)	0.96(0.91–0.99)	0.92(0.88–0.96)	0.94(0.91–0.98)	0.90(0.84–0.98)	0.96(0.91–1.01)	0.98(0.90–1.07)
**Categorical HLI score change**	< = -3	1.16(1.05–1.27)	1.06(0.94–1.20)	1.27(1.10–1.45)	1.10(0.99–1.22)	1.21(0.96–1.54)	1.01(0.86–1.18)	1.23(0.94–1.61)
	-2	1.10(0.99–1.23)	1.10(0.96–1.26)	1.13(0.97–1.31)	1.09(0.97–1.22)	1.14(0.88–1.48)	1.09(0.92–1.29)	1.07(0.78–1.44)
	-1	1.03(0.93–1.13)	1.02(0.90–1.15)	1.08(0.94–1.25)	1.00(0.90–1.11)	1.04(0.81–1.33)	0.99(0.85–1.16)	1.10(0.84–1.45)
	0							
	1	0.99(0.89–1.09)	0.97(0.85–1.10)	1.06(0.92–1.23)	0.97(0.87–1.08)	0.93(0.72–1.20)	0.93(0.79–1.05)	1.09(0.82–1.44)
	2	0.96(0.86–1.07)	0.98(0.85–1.12)	1.02(0.87–1.18)	0.96(0.86–1.08)	0.88(0.66–1.17)	0.95(0.80–1.13)	1.10(0.81–1.49)
	> = 3	0.93(0.84–1.03)	0.92–0.81–1.05)	0.98(0.85–1.14)	0.92(0.82–1.03)	1.00(0.78–1.29)	0.89(0.75–1.05)	1.13(0.85–1.50)
**HLI score change excluding one factor**^**b**^	1-SD increase							
Excluding physical activity	2.0	0.95(0.93–0.98)	0.97(0.93–1.00)	0.94(0.91–0.98)	0.96(0.93–0.99)	0.95(0.88–1.02)	0.97(0.93–1.02)	0.98(0.90–1.06)
Excluding BMI	2.4	0.92(0.89–0.95)	0.95(0.91–0.99)	0.87(0.83–0.91)	0.96(0.93–0.99)	0.98(0.90–1.06)	0.96(0.91–1.01)	0.97(0.88–1.06)
Excluding smoking	2.5	0.95(0.92–0.98)	0.96(0.92–1.00)	0.98(0.94–1.03)	0.94(0.91–0.97)	0.87(0.81–0.94)	0.95(0.91–1.00)	1.00(0.92–1.09)
Excluding alcohol	2.5	0.93(0.90–0.96)	0.97(0.93–1.01)	0.92(0.88–0.96)	0.95(0.92–0.98)	0.89(0.82–0.96)	0.97(0.93–1.02)	0.99(0.91–1.08)
Excluding diet	2.0	0.93(0.90–0.96)	0.96(0.92–1.00)	0.93(0.89–0.97)	0.94(0.91–0.98)	0.90(0.84–0.97)	0.95(0.91–1.00)	0.98(0.90–1.07)
**Single HLI factors**^**c**^	1-unit increase (score 0–4)							
Physical activity score change		0.96(0.94–0.98)	0.98(0.95–1.01)	0.97(0.93–1.00)	0.98(0.95–1.00)	0.94(0.89–0.99)	0.97(0.94–1.01)	1.01(0.95–1.08)
BMI score change		0.98(0.95–1.02)	0.99(0.95–1.03)	1.04(0.99–1.09)	0.96(0.92–0.99)	0.86(0.79–0.94)	0.97(0.92–1.03)	1.03(0.93–1.13)
Smoking score change		0.98(0.93–1.03)	1.02(0.95–1.09)	0.94(0.88–1.00)	1.02(0.96–1.07)	1.08(0.95–1.24)	1.02(0.94–1.11)	0.97(0.84–1.12)
Alcohol score change		0.98(0.95–1.02)	0.94(0.89–0.99)	1.00(0.94–1.06)	0.97(0.93–1.02)	1.06(0.96–1.18)	0.94(0.88–1.00)	0.97(0.86–1.08)
Diet score change		0.99(0.97–1.01)	0.99(0.97–1.02)	0.99(0.97–1.02)	1.00(0.98–1.02)	1.00(0.95–1.05)	1.00(0.96–1.03)	1.00(0.95–1.06)

All models were adjusted for education (years), height (centimetres), HLI score at Q1 (continuous), and calendar year at Q2 (continuous)

^a^Models additionally adjusted for age at menarche (years), menopausal status (premenopausal/postmenopausal), breastfeeding (cumulative months 0, <  = 12, > 12), hormone replacement therapy use (never/former/current), oral contraceptive use (never/ever), parity (0, 1–2, > 2), and history of breast cancer in a first degree relative (yes/no)

^b^Baseline HLI score was adjusted by separately adjusting for HLI score at Q1 excluding the factor in question and the individual factor score at Q1

^c^Mutually adjusted for all single factor HLI score changes and single factor HLI scores at Q1

Alcohol-related cancers including sites: upper aerodigestive [C01-C10], pharynx [C11-C14], esophagus [C15], colorectum [C18-C20], liver [C22-C24], larynx [C32], breast [C50],

Tobacco-related cancers including sites: upper aerodigestive [C01-C10], pharynx [C11-C14], esophagus [C15], stomach [C16], colorectum [C18-C20], liver [C22-C24], pancreas [C25], accessory sinus [C31], larynx [C32], trachea [C33], lung [C34], breast [C50], cervix [C53], ovarian [C56], kidney [C64-C66], bladder [C67], acute myeloid leukemia [C92]

Obesity-related cancers including sites: esophagus [C15], stomach [C16], colorectum [C18-C20], liver [C22-C24], pancreas [C25], breast [C50], uterine [C54-C55], ovarian [C56], kidney [C64-C66], thyroid [C73], multiple myeloma [C90],

Reproductive-related cancers including sites: vulva [C51] vagina [C52], cervix [C53], uterine [C54-C55], ovarian [C56], other female genital organs [C57-C58]

Decreased HLI scores appeared to be statistically significant associated with an increased incidence of lifestyle-related cancer, while increased HLI scores were not (Fig. 2). These results were reflected in the categorical analysis of HLI score change, where decreases of three or more HLI units were associated with a HR of 1.16 (95%CI: 1.05–1.27) and increases of three or more HLI units were associated with a HR of 0.93 (95% CI: 0.84–1.03).

When individual lifestyle factors were excluded one by one, most associations changed less than 5% compared to associations with the HLI including all five lifestyle factors, with some exceptions (Table 2). For tobacco-related cancer incidence, the HR increased by 6.5% to 0.98 (95% CI: 0.94–1.03) when smoking was removed from the HLI and decreased by 5.4% to 0.87 (95% CI: 0.83–0.91) when BMI was removed from the HLI. For reproductive-related cancer incidence, the HR increased by 8.9% to 0.98 (95% CI: 0.90–1.06) when BMI was removed from the HLI and increased by 5.6% to 0.95 (95% CI: 0.88–1.02) when physical activity was removed from the HLI.

In the analysis of individual HLI factors, the HR for physical activity score change (per 1 unit increase) was 0.96 (95% CI: 0.94–0.98) for lifestyle-related cancer incidence and 0.94 (95% CI: 0.89–0.99) for reproductive-related cancer incidence. The HR for BMI score change (per 1 unit increase) was 0.96 (95% CI: 0.92–0.99) for obesity-related cancers and 0.86 (95% CI: 0.79–0.94) for reproductive-related cancers. The HR for smoking score change (per 1 unit increase) was 0.94 (95% CI: 0.88–1.00) for tobacco-related cancer incidence. The HR for alcohol score change (per 1 unit increase) was 0.94 (95% CI: 0.89–0.99) for alcohol-related cancers and 0.94 (95% CI: 0.88–1.00) for breast cancer incidence.

Tests for interaction for age at Q1, age at Q2, and Q1 HLI score category with HLI score change were not significant in adjusted models (all p-values > 0.05). When stratified on enrolment year, there was less than 5% change in estimates compared to the estimate obtained in the main analysis. The estimate for lifestyle-related cancer incidence was unchanged when excluding the first two years of follow-up. There was a null association observed between HLI score change and non-lifestyle-related cancer incidence (HR: 1.02; 95% CI: 0.96, 1.09).

## Discussion

In this study, lifestyle change was assessed by evaluating healthy lifestyle index scores at two timepoints that were on average 7 years apart for 66 233 Norwegian women in the period 1996–2014. Most participants did not report major lifestyle differences between baseline and follow-up, where approximately 38% of participants registered an HLI score improvement. We observed that lifestyle change equivalent to a 1 SD increase in HLI score change was associated with 7% lower incidence for lifestyle-related cancers, 4% lower incidence for alcohol-related cancers, 8% lower incidence for tobacco-related cancer, 6% lower incidence for obesity-related cancers, and 10% lower incidence for reproductive-related cancers. There was a 4% reduced incidence of breast cancer, although the 95% CI for the HR was 0.93 to 1.01. We did not observe an association between HLI score changes and colorectal cancer incidence. When evaluated as group comparisons, major lifestyle worsening corresponding to a decline of three or more HLI score points from baseline to follow-up compared to no HLI score change was associated with a 16% higher risk of overall lifestyle-related cancer. Lifestyle improvement of three or more HLI score points was associated with a 7% lower risk of lifestyle-related cancer, although a null association was also compatible with our data.

In general, lifestyle worsening was both more strongly and more likely associated with the incidence of total lifestyle-related cancers, tobacco-related cancers, obesity-related cancers, and reproductive-related cancers compared to lifestyle improvement. We observed this from results modelling HLI score change as a continuous measure using restricted cubic splines and from modelling HLI score change as group comparisons. However, since there were no clear indications of nonlinearity from the restricted cubic spline models according to visual inspection, this suggests that the linear estimates are robust. Additionally, although we observed the strongest associations for lifestyle worsening, we cannot assert with any confidence that lifestyle improvement is not related to reduced cancer incidence considering the lack of published studies assessing the effects of changes in lifestyle factors in combination.

There are a small number of published studies investigating the effect of changes in lifestyle behaviours combined, as single factors or overall, on cancer incidence. In a study conducted on a large cohort of Swedish women, Botteri et al. [17] observed that those who either improved their lifestyle or maintained their lifestyle had a reduced risk of lifestyle-related cancers compared to those who had consistently poor lifestyle [17]. However, as diet was not included in their HLI and their assessment of lifestyle factor scores was different, more detailed comparison is challenging. A controlled intervention of lifestyle among men at risk for coronary heart disease in Norway observed a 32% risk reduction after 25 years of follow-up [19]. The selected sample and controlled design likely accounted for their stronger estimates compared to ours.

No single lifestyle factor was indicated as solely responsible for the HLI score change associations we observed. Therefore, in combination, changes in physical activity level, BMI, smoking habits, alcohol intake, and diet were related to cancer incidence. In the present study, physical activity score change was the only factor to demonstrate a clear association with lifestyle-related cancer incidence in the single factor analysis. Increasing physical activity level has previously been related to lower cancer incidence among mid-life adults, although the sample was limited to Norwegian men [12]. Oyeyemi et al. [13] observed that, in NOWAC, physical activity level increase was associated with lower colon cancer risk, but not for colorectal cancer, which is consistent with our results. Further, only stable high physical activity levels were associated with lower colon cancer incidence in a large US cohort [28]. Consistent with observations from the Norwegian-Swedish Women’s Lifestyle and Health cohort, we did not observe an association between physical activity level change and breast cancer [29].

Several studies on BMI change – often equated with changes in weight – have identified that weight loss is associated with lower cancer risk [30–33]. However, weight loss has not been shown to influence cancer risk to the same degree or level of certainty as weight gain [7, 32, 34, 35]. Considering that we have identified BMI as an important contributor to the association between continuous HLI score and cancer incidence, the weak associations we observed between lifestyle improvement and lower cancer incidence are consistent with the literature on weight change. Unintentional weight loss as a pre-diagnostic symptom of cancer has been suggested as an explanation for the little to no risk reduction observed among those who lost weight. While the present study did not observe a difference in estimates after conducting sensitivity analysis that excluded the first two years of follow-up, it is possible that unintentional weight loss due to morbid conditions, including cancer, can emerge earlier than two years before diagnosis.

The benefits of smoking cessation for lung cancer [6], head and neck cancer [14], and oesophageal squamous cell carcinoma [36] risk reduction have been widely documented, and are consistent with our results.

We observed that alcohol was an important contributor to the HLI score change associations. Assessed as a single factor, increase in alcohol score change was associated with 6% lower incidence of alcohol-related cancers and breast cancer. A strong positive association between 5-year alcohol consumption increase and breast cancer risk, but not for alcohol reduction was observed among postmenopausal Danish women [18]. This supports our continuous estimate and could add weight to the potential lack of association between overall lifestyle improvement and lower cancer incidence we observed. Unlike our observations, there were no observed associations for alcohol change and incidence of alcohol-related cancers or breast cancer in EPIC [17]. Alcohol cessation has been associated with the lower risks of laryngeal, pharyngeal, and oesophageal cancers [14, 16] supporting our results for alcohol-related cancer. Our observations support the recommendation to reduce alcohol intake for the prevention of several types of cancer.

Diet had the least influence on lifestyle-related cancer incidence compared to other lifestyle factors, given almost unchanged estimates when it was removed from the index and markedly null estimates from the single factor analysis. To our knowledge, studies on dietary change and cancer risk at the individual level do not exist to provide comparison. However, this result is plausible given the lack of convincing evidence between some food groups included in the HLI and cancer incidence as summarised by the WCRF/AICR Continuous Update Project in 2018 [4].

We investigated colorectal cancer incidence as a specific outcome due to its exceptionally high incidence among Norwegian women compared to that of neighbouring and high-income countries [37]. Our study did not observe an association between lifestyle changes, in combination or among individual lifestyle factors, and colorectal cancer incidence. However, in general, the presence of strong and convincing associations between measured risk factors, whether at baseline or at multiple timepoints, and colorectal cancer continue to elude large population-based cohort studies in the Norwegian population [7, 13, 38, 39]. Nevertheless, HLI at baseline and colorectal cancer risk were inversely associated among women in NOWAC [21] and EPIC [40]. This may indicate that, in terms of lifestyle, healthy habits lived from the beginning of adulthood are most important for reducing colorectal cancer risk and/or that the true strength of association is so small that models are underpowered.

Lifestyle changes occurring among Norwegian women in their middle adult years during the period 1996 to 2014 was likely driven by several phenomena, including changes that occurred due to societal shifts in attitudes and availabilities as well as intentional or unintentional individual change. On average, NOWAC women reported increasing physical activity levels, increasing weight, reducing smoking, increasing alcohol intake, and negligible dietary changes on the HLI from baseline until follow-up. Considering this population in its context, we would expect smoking habits to be reduced given increasing tobacco restrictions through the 1990s and 2000s [41]. Weight increase with age, specifically in adult years, is a universal occurrence. Further, national trends have shown that alcohol intake habits among young Norwegian women have been increasing over the past half-century, thus impacting their habits later in adulthood [42]. Due to this, we would expect birth cohorts to undergo systematically different lifestyle changes and for risk to possibly manifest differentially. However, we did not observe different risk estimates for continuous or categorical models between subgroups recruited early or late in the sampling time, despite the wide variation in age, time between baseline and follow-up, and calendar years. This increases our confidence that our estimates reflect risk differences largely attributable to HLI score change.

The findings from our study have major public health relevance. In this study, we provide evidence that overall lifestyle changes among cancer-free women between the ages of 41 and 76 impact the incidence of many cancer types. Importantly, the umbrella grouping of lifestyle-related cancer covers nearly all the most frequent cancers currently diagnosed among adult Norwegian women, including cancers of the breast, lung, colon, and endometrium. To-date, risk differences for lifestyle change have seldom been assessed but are key to making informed policy decisions for how cancer can be prevented in the already adult segment of the population. The importance of having a healthy baseline lifestyle is undeniable. However, our observations indicate that lifestyle changes over a period of five years during adulthood do impact cancer risk, regardless of baseline lifestyle. Further, our results emphasize the importance of avoiding lifestyle worsening. Considering that most Norwegian women in our cohort experienced negative HLI score changes, and thus lifestyle worsening, maintenance of lifestyle should be on the public health agenda.

### Strengths

The minimalism of the HLI enables a broader assessment of lifestyle and an easy method for investigating lifestyle patterns and interaction between single factors. The use of this simple, composite exposure seems to effectively capture an association between lifestyle change and cancer incidence. This supports the use of the HLI as a composite exposure in epidemiological studies given the public health aim to prevent the occurrence of cancer cases.

Additional strengths of this study include its large, nationally representative sample of women in Norway with comprehensive measurements of lifestyle factors and other important characteristics at two timepoints. This data has enabled us to undertake, for the first time, an assessment of the effect of overall lifestyle changes – including physical activity level, BMI, smoking, alcohol, and diet – on cancer incidence. Linkage of participants to the national registries were instrumental in ensuring the follow-up of participants, including cancer case ascertainment, death, and emigration.

### Limitations

There were limitations to the measurement of lifestyle change as a numeric difference between the HLI score measured at two timepoints. Firstly, the data does not inform when the lifestyle change(s) took place beyond recognition of net change between baseline and follow-up. Due to the long latency period of cancers, it is logical that changes occurring closer to baseline, and hence at a younger age, had a greater effect on the outcome compared to changes occurring closer to follow-up, or older age. Not being able to account for these differences likely biased our results to the null. Secondly, changes representing an increase in HLI score in one lifestyle factor concurrent with a decrease in HLI score in another would manifest as a major lifestyle change for the individual, but as a net zero HLI score change. A real example is the known weight gain that follows smoking reduction. Indeed, we observed that weight loss was associated with a higher incidence of tobacco-related cancer. It is therefore possible that our estimates were attenuated in such situations given that both changes are unlikely to represent the same risk compared to no change.

Recall bias is a concern when data is self-reported as it can lead to misclassification error. In NOWAC, height tends to be overestimated and weight tends to be underestimated among participants with overweight and, to a greater extent, obesity [43]. We expect misclassification to have occurred non-differentially across cases and non-cases, thus likely only attenuating rather than biasing our estimates. Under-reporting of unhealthy foods and alcohol has been confirmed in the FFQ used by NOWAC [44]. However, the ranking of individuals’ intake was deemed adequate and the relative validity of the FFQ was observed to be in the same range as observed in other EPIC cohorts [44]. In addition, the FFQs were not identical at baseline and follow-up due to the addition of some food items to the follow-up FFQ that had become relevant for the Norwegian diet after baseline [45]. Although adjustment for energy intake by means of nutrient densities to calculate the diet score accounted for some of these differences, dietary change was likely underestimated. We cannot exclude the presence of residual confounding bias in our risk estimates despite the adjustment of several risk factors. In addition, follow-up time may not have been long enough for the effects of lifestyle changes on cancer development/prevention to accrue.

## Conclusions

This study supports lifestyle intervention as cancer preventive action in the already adult segment of the population. We provide evidence that overall lifestyle changes among cancer-free women between the ages of 41 and 76 impact the incidence of many cancer types. There was a negative dose–response relationship between magnitude of positive lifestyle change and the incidence of overall lifestyle-related cancers, as well as alcohol-related, tobacco-related, obesity-related, and reproductive-related cancers. We observed that underlying this trend was an especially clear association between lifestyle worsening and increased risk compared to stable lifestyle. The prevention of lifestyle worsening, maintenance of healthy lifestyle, and lifestyle improvement, belong on the public health agenda if the predicted trajectory of cancer incidence is to be dismantled.

## Supplementary Information

**Additional file 1.** Questionnaire 1 (baseline), Norwegian Women and Cancer Study (NOWAC).

**Additional file 2.** Questionnaire 2 (follow-up), Norwegian Women and Cancer Study (NOWAC).

**Additional file 3.** Sample flowchart, Norwegian Women and Cancer Study (NOWAC).

**Additional file 4.** Description of healthy lifestyle index (HLI) construction in the Norwegian Women and Cancer Study (NOWAC).

**Additional file 5.** Cancer types and associated International Classification of Disease, tenth revision (ICD-10) codes included in the study.

**Additional file 6.** Distribution of HLI score change across baseline HLI score categories.

**Additional file 7.** Associations between healthy lifestyle index score change and lifestyle-related, alcohol-related, tobacco-related, obesity-related, reproductive-related, breast, and colorectal cancer incidence in the Norwegian Women and Cancer Study (*n* = 44404), complete-case analysis.

**Additional file 8.** Associations between HLI score change modelled with restricted cubic splines and several cancer subgroupings.

## Data Availability

The datasets analysed during the current study are not publicly available due to local and national ethical and security policies but are available from the corresponding author on reasonable request.

## Abbreviations

BMI: Body mass index
EPIC: European prospective investigation into cancer and nutrition
FFQ: Food frequency questionnaire
HLI: Healthy lifestyle index
ICD-10: International Classification of Disease, Version 10
MI: Multiple imputation
NOWAC: Norwegian Women and Cancer Study
Q1: Questionnaire 1 (baseline measurement)
Q2: Questionnaire 2 (follow-up measurement)
