# Supplementary material for: Overall lifestyle changes in adulthood are associated with cancer incidence in the Norwegian Women and Cancer Study (NOWAC) – a prospective cohort study
Source: BMC Public Health. 2023 Apr 3;23:633. doi: 10.1186/s12889-023-15476-3 (PMC10069035; doi:10.1186/s12889-023-15476-3)
Supplement: Supplementary file 1 — Additional file 1. Questionnaire 1 (baseline), Norwegian Women and Cancer Study (NOWAC). [file 12889_2023_15476_MOESM1_ESM.pdf]

KVINNER OG KREFT

KONFIDENSIELT

Hvis du samtykker i å være med, sett kryss for JA i ruten ved siden av. Dersom du ikke ønsker å delta kan du unngå purring ved å sette kryss for NEI og returnere skjemaet i vedlagte svarkonvolutt.
Vi ber deg fylle ut spørreskjemaet så nøye som mulig.

Skjemaet skal leses optisk. Vennligst bruk blå eller sort penn.
Du kan ikke bruke komma, bruk blokkbokstaver.

Med vennlig hilsen
Eiliv Lund
Professor dr. med

Jeg samtykker i å delta i spørreskjemaundersøkelsen JA ☐ NEI ☐

Forhold i oppveksten

I hvilken kommune har du bodd lengre enn ett år?

Kommune: **Alder**

1. Fødested: .... Fra år til ☐☐ år
2. .... Fra ☐☐ år til ☐☐ år
3. .... Fra ☐☐ år til ☐☐ år
4. .... Fra ☐☐ år til ☐☐ år
5. .... Fra ☐☐ år til ☐☐ år
6. .... Fra ☐☐ år til ☐☐ år
7. .... Fra ☐☐ år til ☐☐ år

Kroppstype i 1. klasse. (Sett ett kryss)

☐ veldig tynn ☐ tynn ☐ normal ☐ tykk ☐ veldig tykk

Menstruasjonsforhold

Hvor gammel var du da du fikk menstruasjon første gang? ☐☐

Hvor mange år tok det før menstruasjonen ble regelmessig?

☐ Ett år eller mindre ☐ Mer enn ett år
☐ Aldri ☐ Husker ikke

Har du regelmessig menstruasjon fremdeles?

☐ Ja ☐ Har uregelmessig menstruasjon
☐ Vet ikke (menstruasjon uteblitt pga. sykdom o.l.)
☐ Bruk av hormonpreparat med østrogen
☐ Nei

Hvis Nei;

har den stoppet av seg selv? .... ☐
operert vekk eggstokkene? .... ☐
operert vekk livmoren? .... ☐
annet? .... ☐

Alder da menstruasjonen opphørte? ☐☐

Graviditeter, fødsler og amming

Har du noen gang vært gravid? Ja ☐ Nei ☐

Hvis Ja; fyll ut for hvert barn du har født opplysninger om fødselsår og antall måneder du ammet (fylles også ut for dødfødte eller for barn som er døde senere i livet). Dersom du ikke har født barn, fortsetter du ved neste spørsmål.

Barn	Fødselsår	Antall måneder med amming	Barn	Fødselsår	Antall måneder med amming
1	☐☐	☐☐	5	☐☐	☐☐
2	☐☐	☐☐	6	☐☐	☐☐
3	☐☐	☐☐	7	☐☐	☐☐
4	☐☐	☐☐	8	☐☐	☐☐

Bruk av hormonpreparater med østrogen i overgangsalderen

Har du noen gang brukt østrogentabletter/plaster? .... Ja ☐ Nei ☐

Hvis Ja; hvor mange år har du brukt østrogentabletter/plaster i alt? .... ☐☐

Hvor gammel var du første gang du brukte østrogentabletter/plaster? .... ☐☐

Bruker du tabletter/plaster nå? .... Ja ☐ Nei ☐

Hvor pålitelig anser du kildene nedenfor å være når det gjelder informasjon om østrogenbehandling?

	Lite pålitelig	Pålitelig	Meget pålitelig	Vet ikke/ usikker
Allmenpraktiserende lege	☐	☐	☐	☐
Gynekolog	☐	☐	☐	☐
Apotek	☐	☐	☐	☐
Radio/TV	☐	☐	☐	☐
Ukeblader/aviser	☐	☐	☐	☐
Slekt/venninner	☐	☐	☐	☐

Bruker du soyapreparater mot plager i overgangsalderen? .... Ja ☐ Nei ☐

UTFYLLENDE SPØRSMÅL TIL ALLE SOM HAR BRUKT ELLER BRUKER PREPARATER MED ØSTROGEN I FORM AV TABLETTER ELLER PLASTER.

Hvis du har svart «nei» på spørsmålene om hormonbruk i overgangsalderen, kan du gå videre til spørsmålene under «**P-piller**». Har du svart «ja», ber vi deg om å utdype dette nærmere ved å svare på spørsmålene nedenfor. For hver periode med sammenhengende bruk av samme hormonpreparat håper vi du kan si oss hvor gammel du var da du startet, hvor lenge du brukte det samme hormonpreparatet og navnet på dette. Dersom du har tatt opphold eller skiftet merke, skal du besvare spørsmålene for en ny periode. Dersom du ikke husker navnet på hormonpreparatet sett «usikker». For å hjelpe deg til å huske navnet på hormonpreparatene ber vi deg bruke den vedlagte brosjyre som viser bilder av hormonpreparater som har vært solgt i Norge. Vennligst oppgi også nummer på hormontabletten/plasteret som står i brosjyren.

Periode	Alder ved start	Brukt samme hormontablett/plaster/ Sammenhengende år	måned	Nr.	Hormontablett/ plaster/ (se brosjyre) Navn
1.					
2.					
3.					
4.					
5.					

P-pillebruk

Har du brukt p-piller eller minipiller? .... Ja ☐ Nei ☐

Bruker du p-piller nå? .... Ja ☐ Nei ☐

For p-pillebruk ønsker vi å få vite navnet på p-pillen, årstallet du startet å bruke den og hvor lenge du brukte dette merket sammenhengende. Dersom du har hatt opphold eller skiftet merke start på ny linje. For å hjelpe deg å huske navnet ber vi deg bruke den vedlagte brosjyren. Vennligst oppgi nummeret på p-pillen.

Periode	Alder ved start	Brukt samme hormontablett/plaster/ Sammenhengende år	måned	Nr.	Hormontablett/ plaster/ (se brosjyre) Navn
1.					
2.					
3.					
4.					
5.					
6.					

Hormonspiral

Har du noen gang brukt <u>hormonspiral</u> (Levonova)? .... Ja ☐ Nei ☐

Hvis Ja; hvor mange hele år har du brukt hormonspiral i alt? .... ☐☐

Hvor gammel var du første gang du fikk innsatt <u>hormonspiral</u>? ☐☐

Bruker du <u>hormonspiral</u> nå? .... Ja ☐ Nei ☐

Østrogenpreparat til lokal bruk i skjeden

Har du noen gang brukt østrogenkrem/stikkpille? .... Ja ☐ Nei ☐

Hvis Ja;
bruker du krem/stikkpille nå? .... Ja ☐ Nei ☐

Andre legemidler

Bruker du noen av disse legemidlene daglig nå?

	Ja	Nei
**Fontex, Fluoxetin**	☐	☐
**Cipramil, Citalopram**	☐	☐
**Seroxat, Paroxetin**	☐	☐
**Zoloft**	☐	☐
**Fevarin**	☐	☐
**Cipralex**	☐	☐

Hvis Ja; hvor lenge har du brukt dette legemidlet sammenhengede? Måneder ☐☐ År ☐☐

Har du benyttet noen av disse legemidlene tidligere? Ja ☐ Nei ☐

Hvis Ja; hvor lenge har du benyttet disse legemidlene i alt? År ☐☐

Sykdom

Har du eller har du hatt noen av følgende sykdommer?

	Ja	Nei	Hvis ja: Alder ved start
Kreft	☐	☐	
Høyt blodtrykk	☐	☐	
Hjertesvikt/hjertekrampe	☐	☐	
Hjerteinfarkt	☐	☐	
Slag	☐	☐	
Sukkersyke (diabetes)	☐	☐	
Depresjon (oppsøkt lege)	☐	☐	

Selvopplevd helse

Oppfatter du din egen helse som; (Sett ett kryss)

Meget god ☐ God ☐ Dårlig ☐ Meget dårlig ☐

Røykevaner

Har du i løpet av livet røykt mer enn 100 sigaretter til sammen? .... Ja ☐ Nei ☐

Hvor gammel var du da du tok din første sigarett? ☐☐

Hvis Ja, ber vi deg om å fylle ut for hver aldersgruppe i livet hvor mange sigaretter du i gjennomsnitt røykte pr. dag i den perioden.

Antall sigaretter hver dag

Alder	0	1-4	5-9	10-14	15-19	20-24	25+
10-14	☐	☐	☐	☐	☐	☐	☐
15-19	☐	☐	☐	☐	☐	☐	☐
20-29	☐	☐	☐	☐	☐	☐	☐
30-39	☐	☐	☐	☐	☐	☐	☐
40-49	☐	☐	☐	☐	☐	☐	☐
50+	☐	☐	☐	☐	☐	☐	☐

	Ja	Nei
**Røyker du daglig nå?**	☐	☐
**Røykte noen av dine foreldre når du var barn?**	☐	☐

Hvis Ja, hvor mange sigaretter røykte de til sammen pr. dag? ☐☐

Brystkreft i nærmeste familie

Har noen nære slektninger hatt brystkreft?

	Ja	Nei	Vet ikke	Alder ved start
Datter	☐	☐	☐	☐☐
Mor	☐	☐	☐	☐☐
Søster	☐	☐	☐	☐☐

Mammografiundersøkelse

Har du vært til undersøkelse av brystene med mammografi........ Ja ☐ Nei ☐

Hvis Ja;
hvor gammel var du første gangen? (hele år) .... ☐☐

Hvor mange ganger har du vært undersøkt?

-etter invitasjon fra Mammografiprogrammet........ ☐☐

-etter henvisning fra lege .... ☐☐

-uten henvisning fra lege........ ☐☐

Har du silikoninnlegg i brystene? Ja ☐ Nei ☐

Hvis Ja;
hvor mange år har du hatt det? .... ☐☐

Har du hatt silikoninnlegg tidligere? Ja ☐ Nei ☐

Hvis Ja;
hvorfor fjernet du innlegget? ☐

Fysisk aktivitet

Vi ber deg angi din fysiske aktivitet etter en skala fra svært lite til svært mye. Skalaen nedenfor går fra 1-10. Med fysisk aktivitet mener vi både arbeid i hjemmet og i yrkeslivet, samt trening og annen fysisk aktivitet som turgåing o.l. Sett kryss over det tallet som best angir ditt nivå av fysisk aktivitet.

Alder	Svært lite									Svært mye
14 år	1	2	3	4	5	6	7	8	9	10
30 år	1	2	3	4	5	6	7	8	9	10
I dag	1	2	3	4	5	6	7	8	9	10

Hvor mange timer pr. dag i gjennomsnitt går eller spaserer du utendørs?

	sjelden aldri	mindre enn 1/2 time	1/2-1 time	1-2 timer	mer enn 2 timer
Vinter	☐	☐	☐	☐	☐
Vår	☐	☐	☐	☐	☐
Sommer	☐	☐	☐	☐	☐
Høst	☐	☐	☐	☐	☐

For hver av følgende aktiviteter du deltar i, ber vi deg oppgi hvor mange minutter pr. dag du bruker i gjennomsnitt til hver av aktivitetene.

Fritidsaktivitet	Vinter	Vår	Sommer	Høst
Se på TV........	☐☐☐	☐☐☐	☐☐☐	☐☐☐
Lesing........	☐☐☐	☐☐☐	☐☐☐	☐☐☐
Håndarbeid/hobby	☐☐☐	☐☐☐	☐☐☐	☐☐☐
Hagearbeid........	☐☐☐	☐☐☐	☐☐☐	☐☐☐
Dusj/bad/egenpleie........	☐☐☐	☐☐☐	☐☐☐	☐☐☐

Høyde og vekt

Hvor høy er du? (i hele cm.)........ ☐☐☐

Hvor mye veide du da du var 18 år? (i hele kg.) ☐☐☐

Hvor mye veier du i dag? (i hele kg.) .... ☐☐☐

Kosthold

Påvirker noen av følgende forhold kostholdet ditt?
(sett gjerne flere kryss)

- ☐ Er vegetarianer/veganer
- ☐ Har anoreksi
- ☐ Spiser ikke norsk kost til daglig
- ☐ Har allergi/intoleranse
- ☐ Har bulimi
- ☐ Kronisk sykdom
- ☐ Prøver å gå ned i vekt

Vi er interessert i å få kjennskap til hvordan kostholdet ditt er vanligvis. Kryss av for hvert spørsmål om hvor ofte du i gjennomsnitt siste året har brukt den aktuelle matvaren, og hvor mye du pleier å spise/drikke hver gang.

Hvor mange glass melk drikker du vanligvis av hver type? (Sett ett kryss pr. linje)

	aldri/ sjelden	1-4 pr. uke	5-6 pr. uke	1 pr. dag	2-3 pr. dag	4+ pr. dag
Helmelk (søt, sur)	☐	☐	☐	☐	☐	☐
Lettmelk (søt, sur)	☐	☐	☐	☐	☐	☐
Ekstra lettmelk	☐	☐	☐	☐	☐	☐
Skummet (søt, sur)	☐	☐	☐	☐	☐	☐

Hvor mange kopper kaffe/te drikker du vanligvis av hver sort? (Sett ett kryss for hver linje)

	aldri/ sjelden	1-6 pr. uke	1 pr. dag	2-3 pr. dag	4-5 pr. dag	6-7 pr. dag	8+ pr. dag
Kokekaffe	☐	☐	☐	☐	☐	☐	☐
Traktekaffe	☐	☐	☐	☐	☐	☐	☐
Pulverkaffe	☐	☐	☐	☐	☐	☐	☐
Espresso o.l.	☐	☐	☐	☐	☐	☐	☐
Svart te	☐	☐	☐	☐	☐	☐	☐
Grønn te	☐	☐	☐	☐	☐	☐	☐

Hvor mange glass vann drikker du vanligvis?
(Sett ett kryss for hver linje)

	aldri/ sjelden	1-3 pr. uke	4-6 pr. uke	1 pr. dag	2-3 pr. dag	4+ pr. dag
Springvann	☐	☐	☐	☐	☐	☐
Flaskevann u/kullsyre	☐	☐	☐	☐	☐	☐
Flaskevann m/kullsyre	☐	☐	☐	☐	☐	☐

Hvor mange glass appelsinjuice, saft og brus drikker du vanligvis? (Sett ett kryss for hver linje)

	aldri/ sjelden	1-3 pr. uke	4-6 pr. uke	1 pr. dag	2-3 pr. dag	4+ pr. dag
Appelsinjuice	☐	☐	☐	☐	☐	☐
Saft/brus med sukker	☐	☐	☐	☐	☐	☐
Saft/brus sukkerfri	☐	☐	☐	☐	☐	☐

Hvor ofte spiser du yoghurt (1 beger)? (Sett ett kryss)

☐ Aldri/sjelden ☐ 1 pr. uke ☐ 2-3 pr. uke ☐ 4+ pr. uke

Hvor ofte spiser du kornblanding, havregryn eller müsli? (Sett ett kryss)

☐ Aldri/sjelden ☐ 1-3 pr. uke ☐ 4-6 pr. uke ☐ 1 pr. dag

Hvor mange skiver brød/rundstykker og knekkebrød/skonrokker spiser du vanligvis?
(1/2 rundstykke = 1 brødskive) (Sett ett kryss for hver linje)

	aldri/ sjelden	1-4 pr. uke	5-7 pr. uke	2-3 pr. dag	4-5 pr. dag	6+ pr. dag
**Grovt brød**	☐	☐	☐	☐	☐	☐
**Kneipp/halvfint**	☐	☐	☐	☐	☐	☐
**Fint brød**	☐	☐	☐	☐	☐	☐
**Knekkebrød o.l.**	☐	☐	☐	☐	☐	☐

Nedenfor er det spørsmål om bruk av ulike påleggstyper. Vi spør om hvor mange brødskiver med det aktuelle pålegget du pleier å spise. Dersom du også bruker matvarene i andre sammenhenger enn til brød (f. eks. til vafler, frokostblandinger, grøt), ber vi om at du tar med dette når du besvarer spørsmålene.

På hvor mange brødskiver bruker du? (Sett ett kryss pr. linje)

	0 pr. uke	1-3 pr. uke	4-6 pr. uke	1 pr. dag	2-3 pr. dag	4+ pr. dag
**Syltetøy**	☐	☐	☐	☐	☐	☐
**Brun ost, helfet**	☐	☐	☐	☐	☐	☐
**Brunost, halvfet/mager**	☐	☐	☐	☐	☐	☐
**Hvitost, helfet**	☐	☐	☐	☐	☐	☐
**Hvitost, halvfet/mager**	☐	☐	☐	☐	☐	☐
**Kjøttpålegg, Leverpostei**	☐	☐	☐	☐	☐	☐
**Rekesalat, italiensk o.l.**	☐	☐	☐	☐	☐	☐

På hvor mange brødskiver pr. uke har du i gjennomsnitt siste året spist? (Sett ett kryss pr. linje)

	0 pr. uke	1 pr.uke	2-3 pr.uke	4-6 pr.uke	7-9 pr.uke	10+ pr.uke
**Makrell i tomat, røkt makrell**	☐	☐	☐	☐	☐	☐
**Kaviar**	☐	☐	☐	☐	☐	☐
**Sild/Ansjos**	☐	☐	☐	☐	☐	☐
**Laks (gravet/røkt)**	☐	☐	☐	☐	☐	☐
**Annet fiskepålegg**	☐	☐	☐	☐	☐	☐

Hva slags fett bruker du vanligvis på brødet?
(Sett gjerne flere kryss)

- ☐ Bruker ikke fett på brødet
- ☐ Smør
- ☐ Hard margarin (f. eks. Per, Melange)
- ☐ Myk margarin (f. eks. Soft, Vita, Solsikke)
- ☐ Smørblandet margarin (f.eks. Bremyk)
- ☐ Brelett
- ☐ Lettmargarin (f. eks. Soft light, Letta)
- ☐ Middels lett margarin (f. eks. Olivero, Omega)

Dersom du bruker fett på brødet, hvor tykt lag pleier du smøre på? (En kuvertpakke med margarin veier 12 gram).
(Sett ett kryss)

☐ Skrapet (3 g) ☐ Tynt lag (5 g) ☐ Godt dekket (8 g) ☐ Tykt lag (12 g)

Hvor ofte spiser du frukt? (Sett ett kryss pr. linje)

	aldri/ sjelden	1-3 pr.mnd.	1 pr.uke	2-4 pr.uke	5-6 pr.uke	1 pr.dag	2+ pr. dag
Epler/pærer							
Appelsiner o.l.							
Bananer							
Annen frukt							

Hvor ofte spiser du ulike typer grønnsaker?
(Sett ett kryss pr. linje)

	aldri/ sjelden	1-3 pr.mnd.	1 pr.uke	2 pr.uke	3 pr.uke	4-5 pr.uke	6-7 pr. uke
Gulrøtter							
Kål							
Kålrot							
Brokkoli/blomkål							
Blandet salat							
Tomat							
Grønnsakblanding (frossen)							
Andre grønnsaker							

For de grønnsakene du spiser, kryss av for hvor mye du spiser hver gang. (Sett ett kryss for hver sort)

- gulrøtter	1/2 stk.	1 stk.	1 1/2 stk.	2+ stk.
- kål	1/2 dl	1 dl	1 1/2 dl	2+ dl
- kålrot	1/2 dl	1 dl	1 1/2 dl	2+ dl
- brokkoli/blomkål	1-2 buketter	3-4 buketter	5+ buketter	
- blandet salat	1 dl	2 dl	3 dl	4+ dl
- tomat	1/4	1/2	1	2+
- grønnsakblanding	1/2 dl	1 dl	2 dl	3+ dl

Hvor mange poteter spiser du vanligvis (kokte, stekte, mos)? (Sett ett kryss)

- Spiser ikke/spiser sjelden poteter
- 1-4 pr. uke
- 5-6 pr. uke
- 1 pr. dag
- 2 pr. dag
- 3 pr. dag
- 4+ pr. dag

Hvor ofte bruker du ris og spagetti/makaroni ?
(Sett ett kryss pr. linje)

	aldri/ sjelden	1-3 pr. mnd.	1 pr. uke	2 pr. uke	3+ pr. uke
Ris					
Spagetti, makaroni					

Hvor ofte spiser du grøt ? (Sett ett kryss)

	aldri/ sjelden	1 pr. mnd.	2-3 pr. mnd.	1 pr. uke	2-6 pr. uke	1+ pr. dag
Risengrynsgrøt						
Annen grøt (havre o.l.)						

Fisk

Vi vil gjerne vite hvor ofte du pleier å spise fisk, og ber deg fylle ut spørsmålene om fiskeforbruk så godt du kan. Tilgangen på fisk kan variere gjennom året. Vær vennlig å markere i hvilke årstider du spiser de ulike fiskeslagene.

	aldri/ sjelden	like mye hele året	vintrer	vår	sommer	høst
Torsk, sei, hyse, lyr						
Steinbit, flyndre, uer						
Laks, ørret						
Makrell						
Sild						
Annen fisk						

Med tanke på de periodene av året der du spiser fisk, hvor ofte pleier du å spise følgende?
(Sett ett kryss pr. linje)

	aldri/ sjelden	1 pr. mnd.	2-3 pr. mnd.	1 pr. uke	2+ pr. uke
Kokt torsk, sei, hyse, lyr					
Stekt torsk, sei, hyse, lyr					
Steinbit, flyndre, uer					
Laks, ørret					
Makrell					
Sild					
Annen fisk					

Dersom du spiser fisk, hvor mye spiser du vanligvis pr. gang? (1 skive/stykke = 150 gram)

Kokt fisk (skive)	1	1,5	2	3+
Stekt fisk (stykke)	1	1,5	2	3+

Hvor mange ganger pr. år spiser du fiskeinnmat?
(Sett ett kryss pr. linje)

	0	1-3	4-6	7-9	10+
Rogn					
Fiskelever					

Dersom du spiser fiskelever, hvor mange spiseskjeer pleier du å spise hver gang? (Sett ett kryss)

1	2	3-4	5-6	7+

Hvor ofte bruker du følgende typer fiskemat?
(Sett ett kryss pr. linje)

	aldri/ sjelden	1 pr. mnd.	2-3 pr. mnd.	1 pr. uke	2+ pr. uke
Fiskekaker/pudding/boller					
Plukkfisk/fiskegrateng					
Frityrfisk/fiskepinner					
Andre fiskeretter					

Hvor stor mengde pleier du vanligvis å spise av de ulike rettene? (Sett ett kryss for hver linje)

- fiskekaker/pudding/boller (stk.) (2 fiskeboller=1 fiskekake)	☐ 1	☐ 2	☐ 3	☐ 4+
- plukkfisk, fiskegrateng (dl)	☐ 1-2	☐ 3-4	☐ 5+	
- frityrfisk, fiskepinner (stk.)	☐ 1-2	☐ 3-4	☐ 5-6	☐ 7+

I tillegg til informasjon om fiskeforbruk er det viktig å få kartlagt hvilket tilbehør som blir servert til fisk. Hvor ofte bruker du følgende til fisk? (Sett ett kryss pr. linje)

	aldri/ sjelden	1 pr. mnd.	2-3 pr. mnd.	1 pr. uke	2+ pr. uke
Smeltet smør	☐	☐	☐	☐	☐
Smeltet eller fast margarin/fett	☐	☐	☐	☐	☐
Seterrømme (35%)	☐	☐	☐	☐	☐
Lettrømme (20%)	☐	☐	☐	☐	☐
Saus med fett (hvit/brun)	☐	☐	☐	☐	☐
Saus uten fett (hvit/brun)	☐	☐	☐	☐	☐

For de ulike typene tilbehør du bruker til fisk, vær vennlig å kryss av for hvor mye du vanligvis pleier spise.

- smeltet smør (ss)	☐ 1/2	☐ 1	☐ 2	☐ 3	☐ 4+
- smeltet margasin (ss)	☐ 1/2	☐ 1	☐ 2	☐ 3	☐ 4+
- seterrømme (ss)	☐ 1/2	☐ 1	☐ 2	☐ 3	☐ 4+
- lettrømme (ss)	☐ 1/2	☐ 1	☐ 2	☐ 3	☐ 4+
- saus med fett (dl)	☐ 1/4	☐ 1/2	☐ 3/4	☐ 1	☐ 2+
- saus uten fett (dl)	☐ 1/4	☐ 1/2	☐ 3/4	☐ 1	☐ 2+

Hvor ofte spiser du skalldyr (f. eks. reker, krabbe og skjell)**?** (Sett ett kryss)

☐ Aldri/sjelden ☐ 1 pr. mnd ☐ 2-3 pr. mnd ☐ 1+ pr. uke

Andre matvarer

Hvor ofte spiser du reinkjøtt?

☐ Aldri/sjelden ☐ 1 pr. mnd. ☐ 2-3 pr. mnd. ☐ 1 pr. uke
☐ 2-3 pr. uke ☐ 4+ pr. uke

Hvor ofte spiser du følgende kjøtt- og fjærkreretter?
(Sett ett kryss for hver rett)

	aldri/ sjelden	1 pr.mnd.	2-3 pr.mnd.	1 pr.uke	2+ pr.uke
Steik (okse, svin, får)	☐	☐	☐	☐	☐
Koteletter	☐	☐	☐	☐	☐
Biff	☐	☐	☐	☐	☐
Kjøttkaker, karbonader	☐	☐	☐	☐	☐
Pølser	☐	☐	☐	☐	☐
Gryterett, lapskaus	☐	☐	☐	☐	☐
Pizza med kjøtt	☐	☐	☐	☐	☐
Kylling	☐	☐	☐	☐	☐
Andre kjøttretter	☐	☐	☐	☐	☐

Dersom du spiser følgende retter, oppgi mengden du vanligvis spiser: (Sett ett kryss for hver linje)

- steik (skiver)	☐ 1	☐ 2	☐ 3	☐ 4+
- koteletter (stk.)	☐ 1/2	☐ 1	☐ 1,5	☐ 2+
- kjøttkaker, karbonader (stk.)	☐ 1	☐ 2	☐ 3	☐ 4+
- pølser (stk. à 150g)	☐ 1/2	☐ 1	☐ 1,5	☐ 2+
- gryterett, lapskaus (dl)	☐ 1-2	☐ 3	☐ 4	☐ 5+
- pizza m/kjøtt (stykke à 100 g)	☐ 1	☐ 2	☐ 3	☐ 4+

Hvor mange egg spiser du vanligvis i løpet av en uke?(stekte, kokte, eggerøre, omelett) (Sett ett kryss)

☐ 0 ☐ 1 ☐ 2 ☐ 3-4
☐ 5-6 ☐ 7+

Hvor ofte spiser du iskrem? (til dessert, krone-is osv.)
Sett et kryss for hvor ofte du spiser iskrem om sommeren, og et kryss for resten av året)

	aldri/ sjelden	1-3. pr.	2-3 pr. mnd.	1 pr. uke	2+ pr. uke
-Om sommeren	☐	☐	☐	☐	☐
-Resten av året	☐	☐	☐	☐	☐

Hvor mye is spiser du vanligvis pr. gang? (Sett ett kryss)

☐ 1dl ☐ 2 dl ☐ 3 dl ☐ 4+ dl

Hvor ofte spiser du bakevarer som boller kaker, wienerbrød eller småkaker (Sett ett kryss pr. linje)

	aldri/ sjelden	1-3 mnd.	1 pr. uke	2-3 pr uke	4-6 pr. uke	1+ pr. dag
Gjærbakst (boller)	☐	☐	☐	☐	☐	☐
Wienerbrød, kringle	☐	☐	☐	☐	☐	☐
Kaker (bløtkaker)	☐	☐	☐	☐	☐	☐
Pannekaker	☐	☐	☐	☐	☐	☐
Vafler	☐	☐	☐	☐	☐	☐
Småkaker, kjeks	☐	☐	☐	☐	☐	☐

Hvor ofte spiser du dessert? (Sett ett kryss pr. linje)

	aldri/ sjelden	1-3 mnd.	1 pr. uke	2-3 pr uke	4-6 pr. uke	1+ pr. dag
Pudding sjokolade/karamell	☐	☐	☐	☐	☐	☐
Riskrem, fromasj	☐	☐	☐	☐	☐	☐
Kompott, fruktgrøt, hermetisk frukt	☐	☐	☐	☐	☐	☐
Jorbær (friske, frosne)	☐	☐	☐	☐	☐	☐
Andre bær (friske, frosne)	☐	☐	☐	☐	☐	☐

Hvor ofte spiser du sjokolade? (Sett ett kryss)

	aldri/ sjelden	1-3 mnd.	1 pr. uke	2-3 pr uke	4-6 pr. uke	1+ pr. dag
Mørk sjokolade	☐	☐	☐	☐	☐	☐
Lys sjokolade	☐	☐	☐	☐	☐	☐

Dersom du spiser sjokolade, hvor mye pleier du vanligvis å spise hver gang? Tenk deg størrelsen på en Kvikk-Lunsj sjokolade, og oppgi hvor mye du spiser i forhold til den.

☐ 1/4 ☐ 1/2 ☐ 3/4 ☐ 1 ☐ 1,5 ☐ 2+

Hvor ofte spiser du snacks? (Sett ett kryss)

	aldri/ sjelden	1-3 pr. mnd.	1 pr. uke	2-3 pr. uke	4-6 pr. uke	7+ pr. uke
Potetchips	☐	☐	☐	☐	☐	☐
Peanøtter	☐	☐	☐	☐	☐	☐
Andre nøtter	☐	☐	☐	☐	☐	☐
Annen snacks	☐	☐	☐	☐	☐	☐

Tran og fiskeoljekapsler

Bruker du tran (flytende)? Ja ☐ Nei ☐

Hvis ja; hvor ofte tar du tran?
Sett ett kryss for hver linje.

	aldri/ sjelden	1-3 pr. mnd.	1 pr. uke	2-6 pr. uke	daglig
Om vinteren	☐	☐	☐	☐	☐
Resten av året	☐	☐	☐	☐	☐

Hvor mye tran pleier du å ta hver gang?

☐ 1 ts. ☐ 1/2 ss. ☐ 1+ ss.

Bruker du tranpiller/kapsler? Ja ☐ Nei ☐

Hvis ja; hvor ofte tar du tranpiller/kapsler?
Sett ett kryss for hver linje.

	aldri/ sjelden	1-3 pr. mnd.	1 pr. uke	2-6 pr. uke	daglig
Om vinteren	☐	☐	☐	☐	☐
Resten av året	☐	☐	☐	☐	☐

Hvilken type tranpiller/kapsler bruker du vanligvis, og hvor mange pleier du å ta hver gang?

Navn ____________________ Antall ☐

Bruker du fiskeoljekapsler? (omega-3) Ja ☐ Nei ☐

Hvis ja; hvor ofte tar du fiskeoljekapsler?

aldri/ sjelden	1-3 pr. mnd.	1 pr. uke	2-6 pr. uke	daglig
☐	☐	☐	☐	☐

Hvilken type fiskeoljekapsler bruker du vanligvis, og hvor mange pleier du å ta hver gang?

Navn ____________________ antall ☐

Varm mat

Hvor mange ganger i løpet av en måned spiser du varm mat?

	Antall
Til frokost	☐
Til lunsj	☐
Til middag	☐
Til kvelds	☐

Kosttilskudd

Hvor ofte bruker du kosttilskudd?
(Sett ett kryss pr. linje)

Navn på vitamin/mineraltilskudd:	aldri/ sjelden	1-3 pr. mnd.	1 pr. uke	2-6 pr. uke	daglig
____________	☐	☐	☐	☐	☐
____________	☐	☐	☐	☐	☐
____________	☐	☐	☐	☐	☐

Alkohol

Er du totalavholdskvinne? Ja ☐ Nei ☐

Hvis Nei, hvor ofte og hvor mye drakk du i gjennomsnitt siste året? (Sett ett kryss for hver linje)

	aldri/ sjelden	1 pr. mnd.	2-3 pr. uke	1 pr. uke	2-4 pr. uke	5-6 pr. uke	1+ pr. dag
Øl (1/2 l.)	☐	☐	☐	☐	☐	☐	☐
Vin (glass)	☐	☐	☐	☐	☐	☐	☐
Brennevin (drink)	☐	☐	☐	☐	☐	☐	☐
Likør/Hetvin	☐	☐	☐	☐	☐	☐	☐

Sosiale forhold

Er du: (Sett ett kryss)

☐ gift ☐ samboer ☐ ugift ☐ skilt ☐ enke

Hvor mange års skolegang/yrkesutdannelse har du i alt, ta med folkeskole og ungdomsskole? ☐

Hvor mange personer er det i ditt hushold? ☐

Hvor høy er bruttoinntekten i husholdet pr. år?

under 150.000 kr.	☐	151.000-300.000 kr.	☐
301.000-450.000 kr.	☐	451.000-600.000 kr.	☐
601.000-750.000 kr.	☐	over 750.000 kr.	☐

Hva er din arbeidssituasjon? (sett kryss)

☐ Arbeider heltid ☐ Arbeider deltid ☐ Pensjonist

☐ Hjemmearbeidende ☐ Under utdanning ☐ Uføretrygdet

☐ Under attføring ☐ Arbeidssøkende

Yrke: ____________________

Hvordan var de økonomiske forhold i oppveksten?

☐ Meget gode ☐ Gode

☐ Dårlige ☐ Meget dårlige

Arbeider du utendørs i yrkessammenheng? Ja ☐ Nei ☐

Hvis Ja;
hvor mange timer pr. uke? ....Sommer ....vinter

Solvaner

Får du fregner når du soler deg? .... Ja ☐ Nei ☐

Hvilken øyefarge har du? (sett ett kryss)

☐ brun ☐ grå, grønn eller blanding ☐ blå

Hva er din opprinnelige hårfarge? (sett ett kryss)

☐ mørkbrunt, svart ☐ brun ☐ blond, gul ☐ rød

For å kunne studere effekten av soling på risiko for hudkreft ber vi deg gi opplysninger om hudfarge
Sett ett kryss på det tallet under fargen som best passer din naturlige hudfarge (uten soling)

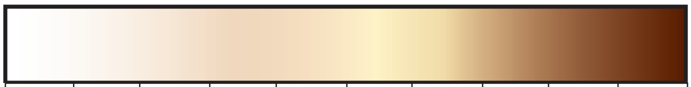

1	2	3	4	5	6	7	8	9	10
☐	☐	☐	☐	☐	☐	☐	☐	☐	☐

Hvor mange ganger pr. år er du blitt forbrent av solen slik at du har fått svie og blemmer med avflassing etterpå? (ett kryss for hver aldersgruppe)

Alder	Aldri	Høyst 1 gang pr. år	2-3 g. pr. år	4-5 g. pr. år	6 eller flere ganger
Før 10 år	☐	☐	☐	☐	☐
10-19 år	☐	☐	☐	☐	☐
20-29 år	☐	☐	☐	☐	☐
30-44 år	☐	☐	☐	☐	☐
45+ år	☐	☐	☐	☐	☐

Hvor mange uker soler du deg pr. år i syden?

Alder	Aldri	1 uke	2-3 uker	4-5 uker	7 uker eller mer
Før 10 år	☐	☐	☐	☐	☐
10-19 år	☐	☐	☐	☐	☐
20-29 år	☐	☐	☐	☐	☐
30-44 år	☐	☐	☐	☐	☐
45+ år	☐	☐	☐	☐	☐
Siste 12 mnd.	☐	☐	☐	☐	☐

Hvor mange uker pr. år soler du deg i Norge eller utenfor syden?

Alder	Aldri	1 uke	2-3 uker	4-5 uker	7 uker eller mer
Før 10 år	☐	☐	☐	☐	☐
10-19 år	☐	☐	☐	☐	☐
20-29 år	☐	☐	☐	☐	☐
30-44 år	☐	☐	☐	☐	☐
45+ år	☐	☐	☐	☐	☐
Siste 12 mnd.	☐	☐	☐	☐	☐

Hvor ofte dusjer eller bader du?

	mer enn 1 g. dagl.	1 g. dagl.	4-6 g. pr. uke	2-3 g. pr. uke	1 g. pr.	2-3 g. pr.uke	sjel-den/ aldri
Med såpe/shampo	☐	☐	☐	☐	☐	☐	☐
Uten såpe/shampo	☐	☐	☐	☐	☐	☐	☐

Når bruker du krem med solfaktor? (sett evt. flere kryss):

☐ i påsken ☐ i Norge eller utenfor syden ☐ solferie i syden
☐ aldri

Hvilken solfaktor bruker du i disse periodene?

	påsken	i Norge eller utenfor syden	solferie i syden
I dag	☐☐	☐☐	☐☐
For 10 år siden	☐☐	☐☐	☐☐

Hvor ofte har du solt deg i solarium?

Alder	Aldri	Sjelden	1 gang pr. mnd.	2 ganger pr. mnd.	3-4 ganger pr. mnd	oftere enn1 gang pr. uke
Før 10 år	☐	☐	☐	☐	☐	☐
10-19 år	☐	☐	☐	☐	☐	☐
20-29 år	☐	☐	☐	☐	☐	☐
30-44 år	☐	☐	☐	☐	☐	☐
45+ år	☐	☐	☐	☐	☐	☐
Siste 12 mnd.	☐	☐	☐	☐	☐	☐

Hvor mange uregelmessige føflekker større enn 5 mm har du sammenlagt på begge beina (fra tærne til lysken)? Tre eksempler på føflekker større enn 5 mm med uregelmessig form er vist i nedenfor.

☐ 0 ☐ 1 ☐ 2-3 ☐ 4-6 ☐ 7-12 ☐ 13-24 ☐ 25+

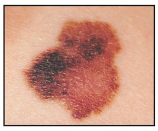

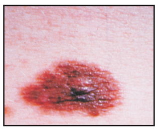

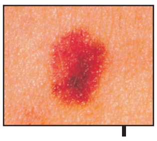

5 mm

Hvor ofte bruker du følgende hudpleiemidler?
(Sett ett kryss pr. linje)

	aldri/ sjelden	1-3 pr.mnd.	1 pr.uke	2-4 pr.uke	5-6 pr.uke	1 pr.dag	2+ pr. dag
Ansiktskrem	☐	☐	☐	☐	☐	☐	☐
Håndkrem	☐	☐	☐	☐	☐	☐	☐
Body lotion	☐	☐	☐	☐	☐	☐	☐
Parfyme	☐	☐	☐	☐	☐	☐	☐

Til slutt vil vi spørre deg om ditt samtykke til å kontakte deg på nytt pr. post. Vi vil hente adressen fra det sentrale personregister.

Ja ☐ Nei ☐

Er du villig til å avgi en blodprøve?

Ja ☐ Nei ☐

Takk for at du ville delta i undersøkelsen
