# Supplementary material for: Overall lifestyle changes in adulthood are associated with cancer incidence in the Norwegian Women and Cancer Study (NOWAC) – a prospective cohort study
Source: BMC Public Health. 2023 Apr 3;23:633. doi: 10.1186/s12889-023-15476-3 (PMC10069035; doi:10.1186/s12889-023-15476-3)
Supplement: Supplementary file 2 — Additional file 2. Questionnaire 2 (follow-up), Norwegian Women and Cancer Study (NOWAC). [file 12889_2023_15476_MOESM2_ESM.pdf]

Høst 2011

KVINNER OG KREFT

Hvis du samtykker i å være med, sett kryss for JA i ruten ved siden av. Dersom du ikke ønsker å delta kan du unngå purring ved å sette kryss for NEI og returnere skjemaet i vedlagte svarkonvolutt. Vi ber deg fylle ut spørreskjemaet så nøye som mulig.

Skjemaet skal leses optisk. Vennligst bruk blå eller sort penn. Du kan ikke bruke komma, forhøy 0,5 til 1. Bruk blokkbokstaver.

Med vennlig hilsen
Eiliv Lund
Professor dr. med

KONFIDENSIELT

47 KK/2011
12384 invit.
300001-330000
8siders - 2.gang

Jeg samtykker i å delta i spørreskjemaundersøkelsen JA ☐ NEI ☐

Menstruasjon og overgangsalder

Har du regelmessig menstruasjon fremdeles?

- ☐ Ja
- ☐ Har uregelmessig menstruasjon
- ☐ Vet ikke (menstruasjon uteblitt pga. sykdom o.l.)
- ☐ Vet ikke (bruker hormonpreparat med østrogen)
- ☐ Nei

Hvis Nei;
har den stoppet av seg selv? ☐
har du operert vekk eggstokkene? ☐
har du operert vekk livmoren? ☐
annet? ☐

Alder da menstruasjonen opphørte ☐☐

Har du eller har du hatt smerter eller ømhet i brystene av minst fem dagers varighet før menstruasjonen? ☐ Ja ☐ Nei
Hvis Ja; i begge brystene? ☐ Ja ☐ Nei
er/var smerten eller ømheten mindre under og etter menstruasjonen? ☐ Ja ☐ Nei
forstyrret plagene ditt sosiale liv, yrkesaktivitet eller privatlivet? ☐ Ja ☐ Nei

Hvor mange år har du hatt slike plager? ☐☐

Bruk av hormonpreparater mot plager i overgangsalderen

Har du noen gang brukt østrogentabletter/plaster? ☐ Ja ☐ Nei
(Gjelder også progestagen/ Tibolon)

Hvis Ja; hvor mange år i alt? ☐☐

Hvor gammel var du første gang du brukte østrogentabletter/plaster? ☐☐

Bruker du tabletter/plaster nå? ☐ Ja ☐ Nei

Utfyllende spørsmål til alle som har brukt preparater med østrogen i form av tabletter eller plaster <u>fra 2003</u> og frem til i dag

Har du svart «ja», ber vi deg utdype dette nærmere ved å svare på spørsmålene nedenfor. For hver periode med sammenhengende bruk av samme hormonpreparat håper vi du kan si oss hvor gammel du var da du startet, hvor lenge du brukte det samme hormonpreparatet og navnet på dette. Dersom du har hatt opphold eller skiftet merke skal du besvare spørsmålene for en ny periode. Dersom du ikke husker navnet på hormonpreparatet, sett «usikker». For å hjelpe deg til å huske navnet på hormonpreparatene ber vi deg bruke vedlagte brosjyre som viser bilder av hormonpreparater som har vært solgt i Norge. Vennligst oppgi også nummer på hormontabletten/plasteret som står i brosjyren.

Periode	Alder ved start	Brukt samme hormontablett/plaster/ sammenhengende fra 2003: antall år	antall mnd.	nr.	Navn på hormontablett/plaster (se brosjyre)
1.					
2.					
3.					
4.					
5.					

Har du eller har du hatt smerter eller ømhet i brystene ved hormonbehandling av plager i overgangsalderen? ☐ Ja ☐ Nei

Hvis Ja;
er/var smerten eller ømheten i begge bryst? ☐ Ja ☐ Nei
forsvinner/forsvant plagene ved stopp av hormonbehandlingen? ☐ Ja ☐ Nei
forstyrrer/forstyrret plagene ditt sosiale liv, yrkesaktivitet eller privatlivet? ☐ Ja ☐ Nei

Byttet du legemiddel? ☐ Ja ☐ Nei
Hvis Ja, ble du bedre? ☐ Ja ☐ Nei

Østrogenpreparat til lokal bruk i skjeden

Har du noen gang brukt østrogen-krem/stikkpille? ☐ Ja ☐ Nei

Hvis Ja; bruker du krem/stikkpille nå? ☐ Ja ☐ Nei

Alternativer til hormonbehandling mot plager i overgangsalderen

Har du noen gang brukt alternativer til hormonbehandling mot plager i overgangsalderen? ☐ Ja ☐ Nei

Hvis Ja; har du brukt noen av følgende:

Soyatilskudd ☐ Ja ☐ Nei

Preparatnavn ____________

Andre tilskudd for overgangsplager ☐ Ja ☐ Nei

Preparatnavn ____________

Andre legemidler enn hormoner ☐ Ja ☐ Nei

Preparatnavn ____________

Akupunktur ☐ Ja ☐ Nei

Homeopati ☐ Ja ☐ Nei

Avspenningsteknikk/trening ☐ Ja ☐ Nei

Andre alternativer, spesifiser: ☐ Ja ☐ Nei

____

Sykdom

Har du eller har du hatt noen av følgende sykdommer? (sett ett eller flere kryss)

	Ja	Nei	Hvis ja: Alder ved start
Kreft	☐	☐	
Høyt blodtrykk	☐	☐	
Hjertesvikt/hjertekrampe	☐	☐	
Hjerteinfarkt	☐	☐	
Slag	☐	☐	
Depresjon (oppsøkt lege)	☐	☐	
Hypothyreose/lavt stoffskifte	☐	☐	
Sukkersyke (diabetes)	☐	☐	

Hvis ja på sukkersyke, hvilken type:

☐ Type 1 ☐ Aldersdiabetes ☐ Svangerskap

Behandles du i dag med (sett ett eller flere kryss):

☐ Insulin ☐ Legemidler ☐ Kost

For følgende tilstander ber vi deg krysse av for hvilket år tilstanden oppsto første gang

	før 04	04	05	06	07	08	09	10	11
Muskelsmerter (myalgi)	☐	☐	☐	☐	☐	☐	☐	☐	☐
Fibromyalgi/Fibrositt	☐	☐	☐	☐	☐	☐	☐	☐	☐
Kronisk tretthetssyndrom	☐	☐	☐	☐	☐	☐	☐	☐	☐
Ryggsmerter ukjent årsak	☐	☐	☐	☐	☐	☐	☐	☐	☐
Nakkeslengskade	☐	☐	☐	☐	☐	☐	☐	☐	☐
Osteoporose (benskjørhet)	☐	☐	☐	☐	☐	☐	☐	☐	☐
**Brudd**									
Underarmen (håndledd)	☐	☐	☐	☐	☐	☐	☐	☐	☐
Lårhalsen	☐	☐	☐	☐	☐	☐	☐	☐	☐
Ryggvirvel (kompresjon)	☐	☐	☐	☐	☐	☐	☐	☐	☐

Selvopplevd helse

Oppfatter du din egen helse som? (Sett ett kryss)

☐ Meget god ☐ God ☐ Dårlig ☐ Meget dårlig

Høyde og vekt

Hvor høy er du i dag? (i hele cm)

Hvor mye veier du i dag? (i hele kg)

Røykevaner

Har du i løpet av livet røykt mer enn 100 sigaretter til sammen? ☐ Ja ☐ Nei

Hvis Ja, ber vi deg fylle ut for perioden 2003–2011 hvor mange sigaretter du i gjennomsnitt røykte pr. dag.

Antall sigaretter pr. dag

	0	1–4	5–9	10–14	15–19	20–24	25+
2003–2007	☐	☐	☐	☐	☐	☐	☐
2008–2011	☐	☐	☐	☐	☐	☐	☐

Hvor gammel var du da du tok din første sigarett?

Røyker du daglig nå? ☐ Ja ☐ Nei

Hvis Nei, hvor gammel var du da du sluttet?

Røyker du av og til nå? ☐ Ja ☐ Nei

Røykte noen av dine foreldre da du var barn? ☐ Ja ☐ Nei

Hvis Ja, hvor mange sigaretter røykte de til sammen pr. dag? (Antall)

Fysisk aktivitet

Vi ber deg angi din fysiske aktivitet etter en s
svært lite til svært mye ved 14 års alder, ved
og i dag. Skalaen nedenfor går fra 1–10. Me
aktivitet mener vi både arbeid i hjemmet og
samt trening og annen fysisk aktivitet som tu

Alder	Svært lite						
14 år	1	2	3	4	5	6	7
50 år	1	2	3	4	5	6	7
I dag	1	2	3	4	5	6	7

Vi er interessert i informasjon om ulike form
aktivitet i dagliglivet. Spørsmålene gjelder t
brukt på fysisk aktivitet de siste 7 dagene.
svar på alle spørsmålene uansett hvor fysisk
Tenk på aktiviteter du gjør på jobb, som en
og hagearbeid, for å komme deg fra et ste
og aktiviteter på fritiden (rekreasjon, mosjo

Tenk på alle svært anstrengende aktiviteter
drevet med de siste 7 dagene. Svært anstr
aktivitet er aktivitet som krever hard innsats
til å puste mye mer enn vanlig. Ta bare me
som varer minst 10 minutter i strekk.

1. Hvor mange dager i løpet av de siste 7
drevet med meget anstrengende aktivite
løft, gravearbeid, aerobics, løp eller rask

Dager i uken

☐ Ingen meget anstrengende aktivitet *Gå*

2. Hvor lang tid brukte du vanligvis på svært anstrengende aktivitet en av disse dagene? timer pr. dag

Tenk på all middels anstrengende aktivitet
drevet med de siste 7 dagene. Middels ans
aktivitet er aktivitet som krever moderat inn
får deg til å puste litt mer enn vanlig. Ta ba
aktiviteter som varer minst 10 minutter i str

3. Hvor mange dager i løpet av de siste 7
du drevet med middels anstrengende fy
som å bære lette ting, jogge eller sykle
tempo? Ikke ta med gange.

Dager i uken

☐ Ingen *Gå til spørsmål 5*

4. Hvor lang tid brukte du vanlig-vis på middels anstrengende fysisk aktivitet på en av disse dagene? timer pr. dag

Fysisk aktivitet

Vi ber deg angi din fysiske aktivitet etter en skala fra svært lite til svært mye ved 14 års alder, ved 50 års alder og i dag. Skalaen nedenfor går fra 1–10. Med fysisk aktivitet mener vi både arbeid i hjemmet og i yrkeslivet samt trening og annen fysisk aktivitet som turgåing ol.

Alder	Svært lite									Svært mye
14 år	1	2	3	4	5	6	7	8	9	10
50 år	1	2	3	4	5	6	7	8	9	10
I dag	1	2	3	4	5	6	7	8	9	10

Vi er interessert i informasjon om ulike former for fysisk aktivitet i dagliglivet. Spørsmålene gjelder tiden du har brukt på fysisk aktivitet de siste 7 dagene. Vennligst svar på alle spørsmålene uansett hvor fysisk aktiv du er. Tenk på aktiviteter du gjør på jobb, som en del av hus- og hagearbeid, for å komme deg fra et sted til et annet og aktiviteter på fritiden (rekreasjon, mosjon og sport).

Tenk på alle svært anstrengende aktiviteter du har drevet med de siste 7 dagene. Svært anstrengende aktivitet er aktivitet som krever hard innsats og får deg til å puste mye mer enn vanlig. Ta bare med aktiviteter som varer minst 10 minutter i strekk.

1. Hvor mange dager i løpet av de siste 7 dager har du drevet med meget anstrengende aktivitet som tunge løft, gravearbeid, aerobics, løp eller rask sykling?

   Dager i uken ☐

   ☐ Ingen meget anstrengende aktivitet *Gå til spørsmål 3*

2. Hvor lang tid brukte du vanligvis på svært anstrengende aktivitet en av disse dagene? ... timer pr. dag ☐ minutter pr. dag ☐ Vet ikke ☐

Tenk på all middels anstrengende aktivitet du har drevet med de siste 7 dagene. Middels anstrengende aktivitet er aktivitet som krever moderat innsats og får deg til å puste litt mer enn vanlig. Ta bare med aktiviteter som varer minst 10 minutter i strekk.

3. Hvor mange dager i løpet av de siste 7 dagene har du drevet med middels anstrengende fysisk aktivitet som å bære lette ting, jogge eller sykle i moderat tempo? Ikke ta med gange.

   Dager i uken ☐

   ☐ Ingen *Gå til spørsmål 5*

4. Hvor lang tid brukte du vanligvis på middels anstrengende fysisk aktivitet på en av disse dagene? ... timer pr. dag ☐ minutter pr. dag ☐ Vet ikke ☐

Tenk på tiden du har brukt på å gå de siste 7 dagene. Dette inkluderer gange på jobb og hjemme, gange fra ett sted til et annet eller gange som du gjør på tur eller som trening på fritiden.

5. Hvor mange dager i løpet av de siste 7 dagene gikk du i minst 10 minutter i strekk?

   Dager i uken ☐

   ☐ Ingen *Gå til spørsmål 7*

6. Hvor lang tid brukte du vanligvis på å gå en av disse dagene? ... timer pr. dag ☐ minutter pr. dag ☐ Vet ikke ☐

Tenk på all tid du har tilbrakt sittende på ukedagene i løpet av de siste 7 dagene. Inkluder tid du har brukt på å sitte på jobb, hjemme, på kurs og på fritiden. Dette kan tilsvare tiden du sitter ved et arbeidsbord, hos venner, mens du leser eller sitter eller ligger for å se på TV.

7. Hvor lang tid brukte du på å sitte en vanlig hverdag i løpet av de siste 7 dagene? ... timer pr. dag ☐ minutter pr. dag ☐ Vet ikke ☐

Brystkreft i nærmeste familie

Har noen nære slektninger hatt brystkreft?

	Ja	Nei	Vet ikke	Alder ved start
Datter	☐	☐	☐	☐
Mor	☐	☐	☐	☐
Søster	☐	☐	☐	☐

Mammografiundersøkelse

Har du vært til undersøkelse av brystene med mammografi ... ☐ Ja ☐ Nei

Hvis Ja, hvor gammel var du? (hele år) ... første gang ☐ siste gang ☐

Hvor mange ganger har du vært undersøkt?

-etter invitasjon fra Kreftregisteret/ Det nasjonale mammografiprogrammet ... ☐

-etter henvisning fra lege ... ☐

-uten henvisning fra lege ... ☐

- som del av egen forsikring/ gjennom arbeidsplass ... ☐

- gjennom frivillige organisasjoner ... ☐

Kosthold

Vi er interessert i å få kjennskap til hvordan kostholdet ditt er vanligvis. Kryss av for hvert spørsmål om hvor ofte du i gjennomsnitt siste året har brukt den aktuelle matvaren, og hvor mye du pleier å spise/drikke hver gang.

Drikke

Hvor mange glass melk drikker du vanligvis av hver type? (Sett ett kryss pr. linje)

	aldri/ sjelden	1–4 pr. uke	5–6 pr. uke	1 pr. dag	2–3 pr. dag	4+ pr.dag
Helmelk (søt, sur)	☐	☐	☐	☐	☐	☐
Lettmelk (søt, sur)	☐	☐	☐	☐	☐	☐
Ekstra lettmelk	☐	☐	☐	☐	☐	☐
Skummet (søt, sur)	☐	☐	☐	☐	☐	☐

Hvor mange kopper kaffe/te drikker du vanligvis av hver sort? (Sett ett kryss for hver linje)

	aldri/ sjelden	1–6 pr. uke	1 pr. dag	2–3 pr. dag	4–5 pr.dag	6–7 pr.dag	8+ pr.dag
Kokekaffe, presskanne	☐	☐	☐	☐	☐	☐	☐
Traktekaffe	☐	☐	☐	☐	☐	☐	☐
Espresso	☐	☐	☐	☐	☐	☐	☐
Latte	☐	☐	☐	☐	☐	☐	☐
Pulverkaffe	☐	☐	☐	☐	☐	☐	☐
Svart te	☐	☐	☐	☐	☐	☐	☐
Grønn te	☐	☐	☐	☐	☐	☐	☐

Bruker du følgende i kaffe

Sukker *(ikke kunstig søtstoff)*	☐ Ja	☐ Nei
Melk eller fløte	☐ Ja	☐ Nei

Bruker du følgende i te

Sukker *(ikke kunstig søtstoff)*	☐ Ja	☐ Nei
Melk eller fløte	☐ Ja	☐ Nei

Hvor mange glass vann drikker du vanligvis?
(Sett ett kryss for hver linje)

	aldri/ sjelden	1–6 pr. uke	1 pr. dag	2–3 pr. dag	4–5 pr.dag	6–7 pr.dag	8+ pr.dag
Springvann/ flaskevann	☐	☐	☐	☐	☐	☐	☐

Hvor mange glass juice, saft og brus drikker du vanligvis? (Sett ett kryss for hver linje)

	aldri/ sjelden	1–3 pr. uke	4–6 pr. uke	1 pr. dag	2–3 pr. dag	4+ pr.dag
Appelsinjuice	☐	☐	☐	☐	☐	☐
Annen juice	☐	☐	☐	☐	☐	☐
Saft/brus med sukker	☐	☐	☐	☐	☐	☐
Saft/brus sukkerfri	☐	☐	☐	☐	☐	☐

Yoghurt/kornblanding

Hvor ofte spiser du yoghurt (1 beger)? (Sett ett kryss)

☐ Aldri/sjelden ☐ 1–3 pr. uke
☐ 4–6 pr. uke ☐ 1 + pr. dag

Hvor ofte spiser du kornblanding, havregryn eller müsli? (Sett ett kryss)

☐ Aldri/sjelden ☐ 1–3 pr. uke
☐ 4–6 pr. uke ☐ 1 + pr. dag

Brødmat

Hvor mange skiver brød/rundstykker og knekkebrød/skonrokker spiser du vanligvis?
(½ rundstykke = 1 brødskive) (Sett ett kryss for hver linje)

	aldri/ sjelden	1–4 pr. uke	5–7 pr. uke	2–3 pr. dag	4–5 pr. dag	6+ pr.dag
Grovt brød	☐	☐	☐	☐	☐	☐
Kneipp/halvfint	☐	☐	☐	☐	☐	☐
Fint brød/baguett	☐	☐	☐	☐	☐	☐
Knekkebrød o.l.	☐	☐	☐	☐	☐	☐

Nedenfor er det spørsmål om bruk av ulike påleggstyper. Vi spør om hvor mange brødskiver med det aktuelle pålegget du pleier å spise. Dersom du også bruker matvarene i andre sammenhenger enn til brød (f. eks. til vafler, frokostblandinger, grøt), ber vi om at du tar med dette når du besvarer spørsmålene.

På hvor mange brødskiver bruker du?
(Sett ett kryss pr. linje)

	aldri/ sjelden	1–3 pr. uke	4–6 pr. uke	1 pr. dag	2–3 pr. dag	4+ pr. dag
Syltetøy	☐	☐	☐	☐	☐	☐
Brunost, helfet	☐	☐	☐	☐	☐	☐
Brunost, halvfet/mager	☐	☐	☐	☐	☐	☐
Hvitost, helfet	☐	☐	☐	☐	☐	☐
Hvitost, halvfet/mager	☐	☐	☐	☐	☐	☐
Rekesalat, italiensk o.l.	☐	☐	☐	☐	☐	☐

Kjøttpålegg
(Sett ett kryss pr. linje)

	aldri/ sjelden	1–3 pr. uke	4–6 pr. uke	1 pr. dag	2–3 pr. dag	4+ pr. dag
Leverpostei	☐	☐	☐	☐	☐	☐
Magert (kokt skinke o.l.)	☐	☐	☐	☐	☐	☐
Fett (salami, fenalår o.l.)	☐	☐	☐	☐	☐	☐

På hvor mange brødskiver pr. uke har du i gjennomsnitt siste året spist? (Sett ett kryss pr. linje)

	aldri/ sjelden	1 pr. uke	2–3 pr. uke	4–6 pr. uke	7–9 pr. uke	10+ pr. uke
Makrell i tomat, røkt makrell	☐	☐	☐	☐	☐	☐
Kaviar	☐	☐	☐	☐	☐	☐
Sild/Ansjos	☐	☐	☐	☐	☐	☐
Laks *(gravet/røkt)*	☐	☐	☐	☐	☐	☐
Annet fiskepålegg	☐	☐	☐	☐	☐	☐

Dersom du bruker fett på brødet, hvor tykt lag pleier du å smøre på? (En kuvertpakke med margarin veier 12 gram). (Sett ett kryss)

☐ Skrapet *(3 g)* ☐ Tynt lag *(5 g)*
☐ Godt dekket *(8 g)* ☐ Tykt lag *(12 g)*

Hva slags fett bruker du vanligvis på brø
(Sett gjerne flere kryss)

☐ Bruker ikke fett på brødet
☐ Smør
☐ Hard margarin *(f. eks. Melange)*
☐ Myk margarin *(f. eks. Soft, Vita)*
☐ Smørblandet margarin *(f.eks. Bremyk)*
☐ Brelett
☐ Lettmargarin *(f. eks. Soft light, Vita Lett)*
☐ Margarin med olivenolje *(f. eks. Brelett olive*

Frukt og grønnsaker

Hvor ofte spiser du frukt? (Sett ett kryss pr. li

	aldri/ sjelden	1–3 pr. mnd	1 pr. uke	2–4 pr. uke	5–6 pr. u
Epler/pærer	☐	☐	☐	☐	☐
Appelsiner o.l.	☐	☐	☐	☐	☐
Bananer	☐	☐	☐	☐	☐
Annen frukt	☐	☐	☐	☐	☐

Hvor ofte spiser du kokt potet? (Sett ett kry

	aldri/ sjelden	1–4 pr. mnd	5–6 pr. uke	1 pr. dag	2 pr dag
Kokt	☐	☐	☐	☐	☐

Hvor mange poteter spiser du hver gang
(Sett ett kryss)

☐ 0 ☐ 1 ☐ 2 ☐ 3–4 ☐ 5–6 ☐

Hvor ofte spiser du stekt, fritert eller mos
(Sett ett kryss pr. linje)

	aldri/ sjelden	1–4 pr. mnd	5–6 pr. uke	1 pr. dag	2 pr dag
Stekt, fritert, most	☐	☐	☐	☐	☐

Hvor ofte spiser du ulike typer grønnsake
(Sett ett kryss pr. linje)

	aldri/ sjelden	1–3 pr. mnd.	1 pr. uke	2 pr. uke	3 pr uke
Gulrøtter	☐	☐	☐	☐	☐
Kål	☐	☐	☐	☐	☐
Kålrot	☐	☐	☐	☐	☐
Brokkoli/blomkål	☐	☐	☐	☐	☐
Blandet salat	☐	☐	☐	☐	☐
Tomat	☐	☐	☐	☐	☐
Grønnsakblanding	☐	☐	☐	☐	☐
Løk	☐	☐	☐	☐	☐
Bønner	☐	☐	☐	☐	☐
Erter	☐	☐	☐	☐	☐
Andre grønnsaker	☐	☐	☐	☐	☐

Hva slags fett bruker du vanligvis på brødet?
(Sett gjerne flere kryss)

- ☐ Bruker ikke fett på brødet
- ☐ Smør
- ☐ Hard margarin *(f. eks. Melange)*
- ☐ Myk margarin *(f. eks. Soft, Vita)*
- ☐ Smørblandet margarin *(f.eks. Bremyk)*
- ☐ Brelett
- ☐ Lettmargarin *(f. eks. Soft light, Vita Lett)*
- ☐ Margarin med olivenolje *(f. eks. Brelett oliven, Soft oliven)*

Frukt og grønnsaker

Hvor ofte spiser du frukt? (Sett ett kryss pr. linje)

	aldri/ sjelden	1–3 pr. mnd	1 pr. uke	2–4 pr. uke	5–6 pr. uke	1 pr.dag	2+ pr.dag
Epler/pærer	☐	☐	☐	☐	☐	☐	☐
Appelsiner o.l.	☐	☐	☐	☐	☐	☐	☐
Bananer	☐	☐	☐	☐	☐	☐	☐
Annen frukt	☐	☐	☐	☐	☐	☐	☐

Hvor ofte spiser du kokt potet? (Sett ett kryss pr. linje)

	aldri/ sjelden	1–4 pr. mnd	5–6 pr. uke	1 pr. dag	2 pr. dag
Kokt	☐	☐	☐	☐	☐

Hvor mange poteter spiser du hver gang?
(Sett ett kryss)

☐ 0 ☐ 1 ☐ 2 ☐ 3–4 ☐ 5–6 ☐ 7+

Hvor ofte spiser du stekt, fritert eller most potet
(Sett ett kryss pr. linje)

	aldri/ sjelden	1–4 pr. mnd	5–6 pr. uke	1 pr. dag	2 pr. dag
Stekt, fritert, most	☐	☐	☐	☐	☐

Hvor ofte spiser du ulike typer grønnsaker?
(Sett ett kryss pr. linje)

	aldri/ sjelden	1–3 pr. mnd.	1 pr. uke	2 pr. uke	3 pr. uke	4–5 pr. uke	6–7 pr. uke
Gulrøtter	☐	☐	☐	☐	☐	☐	☐
Kål	☐	☐	☐	☐	☐	☐	☐
Kålrot	☐	☐	☐	☐	☐	☐	☐
Brokkoli/blomkål	☐	☐	☐	☐	☐	☐	☐
Blandet salat	☐	☐	☐	☐	☐	☐	☐
Tomat	☐	☐	☐	☐	☐	☐	☐
Grønnsakblanding	☐	☐	☐	☐	☐	☐	☐
Løk	☐	☐	☐	☐	☐	☐	☐
Bønner	☐	☐	☐	☐	☐	☐	☐
Erter	☐	☐	☐	☐	☐	☐	☐
Andre grønnsaker	☐	☐	☐	☐	☐	☐	☐

For de grønnsakene du spiser, kryss av for hvor mye du spiser hver gang. (Sett ett kryss for hver sort)

Gulrøtter	☐ ½ stk	☐ 1 stk	☐ 1 ½ stk	☐ 2+ stk.
Kål	☐ ½ dl	☐ 1 dl	☐ 1 ½ dl	☐ 2+ dl
Kålrot	☐ ½ dl	☐ 1 dl	☐ 1 ½ dl	☐ 2+ dl
Brokkoli/blomkål	☐ 1–2 buketter	☐ 3–4 buketter	☐ 5+ buketter	
Blandet salat	☐ 1 dl	☐ 2 dl	☐ 3dl	☐ 4+ dl
Tomat	☐ 1/4 stk	☐ ½ stk	☐ 1 stk	☐ 2+ stk.
Grønnsakblanding	☐ ½ dl	☐ 1 dl	☐ 2 dl	☐ 3+ dl
Bønner	☐ 1–2 ss	☐ 3–4 ss	☐ 5–6 ss	☐ 7+ ss
Erter	☐ 1–2 ss	☐ 3–4 ss	☐ 5–6 ss	☐ 7+ ss

Ris, spaghetti, grøt, suppe

Hvor ofte bruker du ris og spaghetti/makaroni?
(Sett ett kryss pr. linje)

	aldri/ sjelden	1–3 pr. mnd	1 pr. uke	2 pr. uke	3+ pr. uke
Ris	☐	☐	☐	☐	☐
Spagetti, makaroni, nudler	☐	☐	☐	☐	☐

Hvor ofte spiser du grøt ? (Sett ett kryss pr. linje)

	aldri/ sjelden	1 pr. mnd	2–3 pr. mnd	1 pr. uke	2–6 pr. uke	1+ pr. dag
Risengrynsgrøt	☐	☐	☐	☐	☐	☐
Annen grøt (havre o.l.)	☐	☐	☐	☐	☐	☐

Hvor ofte spiser du suppe? (Sett ett kryss pr. linje)

	aldri/ sjelden	1–3 pr. mnd	1 pr. uke	2 pr. uke	3+ pr. uke
Som hovedrett	☐	☐	☐	☐	☐
Som forrett, lunsj eller kveldsmat	☐	☐	☐	☐	☐

Fisk

Vi vil gjerne vite hvor ofte du pleier å spise fisk, og ber deg fylle ut spørsmålene om fiskeforbruk så godt du kan. Tilgangen på fisk kan variere gjennom året. Vær vennlig å markere i hvilke årstider du spiser de ulike fiskeslagene.

	aldri/ sjelden	like mye hele året	vinter	vår	sommer	høst
Torsk, sei, hyse, lyr	☐	☐	☐	☐	☐	☐
Steinbit, flyndre, uer	☐	☐	☐	☐	☐	☐
Laks, ørret	☐	☐	☐	☐	☐	☐
Makrell	☐	☐	☐	☐	☐	☐
Sild	☐	☐	☐	☐	☐	☐
Annen fisk	☐	☐	☐	☐	☐	☐

Med tanke på de periodene av året der du spiser fisk, hvor ofte pleier du å spise følgende til middag? (Sett ett kryss pr. linje)

	aldri/ sjelden	1 pr. mnd.	2–3 pr. mnd	1 pr. uke	2+ pr. uke
Kokt torsk, sei, hyse, lyr	☐	☐	☐	☐	☐
Stekt torsk, sei, hyse, lyr	☐	☐	☐	☐	☐
Steinbit, flyndre, uer	☐	☐	☐	☐	☐
Laks, ørret	☐	☐	☐	☐	☐
Makrell	☐	☐	☐	☐	☐
Sild	☐	☐	☐	☐	☐
Annen fisk	☐	☐	☐	☐	☐

Dersom du spiser fisk, hvor mye spiser du vanligvis pr. gang? (1 skive/stykke = 150 gram)

Kokt fisk *(skive)* ☐ 1 ☐ 1,5 ☐ 2 ☐ 3+
Stekt fisk *(stykke)* ☐ 1 ☐ 1,5 ☐ 2 ☐ 3+

Hvor mange ganger pr. år spiser du fiskeinnmat? (Sett ett kryss pr. linje)

	0	1–3	4–6	7–9	10+
Rogn	☐	☐	☐	☐	☐
Fiskelever	☐	☐	☐	☐	☐

Dersom du spiser fiskelever, hvor mange spiseskjeer pleier du å spise hver gang? (Sett ett kryss)

☐ 1 ☐ 2 ☐ 3–4 ☐ 5–6 ☐ 7+

Hvor ofte bruker du følgende typer fiskemat? (Sett ett kryss pr. linje)

	aldri/ sjelden	1 pr. mnd.	2–3 pr. mnd	1 pr. uke	2+ pr. uke
Fiskekaker/pudding/boller	☐	☐	☐	☐	☐
Plukkfisk/fiskegrateng	☐	☐	☐	☐	☐
Frityrfisk/fiskepinner	☐	☐	☐	☐	☐
Andre fiskeretter	☐	☐	☐	☐	☐

Hvor stor mengde pleier du vanligvis å spise av de ulike rettene? (Sett ett kryss for hver linje)

Fiskekaker/pudding/boller *(stk.) (2 fiskeboller=1 fiskekake)* ☐ 1 ☐ 2 ☐ 3 ☐ 4+
Plukkfisk, fiskegrateng *(dl)* ☐ 1–2 ☐ 3–4 ☐ 5+
Frityrfisk, fiskepinner *(stk.)* ☐ 1–2 ☐ 3–4 ☐ 5–6 ☐ 7+

I tillegg til informasjon om fiskeforbruk er det viktig å få kartlagt hvilket tilbehør som blir servert til fisk. Hvor ofte bruker du følgende til fisk?

	aldri/ sjelden	1 pr. mnd.	2–3 pr. mnd	1 pr. uke	2+ pr. uke
Smeltet/fast smør	☐	☐	☐	☐	☐
Smeltet/fast margarin	☐	☐	☐	☐	☐
Seterrømme *(35%)*	☐	☐	☐	☐	☐
Lettrømme *(20%)*	☐	☐	☐	☐	☐
Saus med fett *(hvit/brun)*	☐	☐	☐	☐	☐
Saus uten fett *(hvit/brun)*	☐	☐	☐	☐	☐

For de ulike typene tilbehør du bruker til fisk, vær vennlig å kryss av for hvor mye du vanligvis pleier å spise.

Smeltet/fast smør *(ss)* ☐ ½ ☐ 1 ☐ 2 ☐ 3 ☐ 4+
Smeltet/fast margarin *(ss)* ☐ ½ ☐ 1 ☐ 2 ☐ 3 ☐ 4+
Seterrømme *(ss)* ☐ ½ ☐ 1 ☐ 2 ☐ 3 ☐ 4+
Lettrømme *(ss)* ☐ ½ ☐ 1 ☐ 2 ☐ 3 ☐ 4+
Saus med fett *(dl)* ☐ ¼ ☐ ½ ☐ ¾ ☐ 1 ☐ 2+
Saus uten fett *(dl)* ☐ ¼ ☐ ½ ☐ ¾ ☐ 1 ☐ 2+

Hvor ofte spiser du skalldyr (f. eks. reker, krabbe og skjell)? (Sett ett kryss)

☐ Aldri/sjelden ☐ 1 pr. mnd
☐ 2–3 pr. mnd ☐ 1+ pr. uke

Hva bruker du vanligvis å steke i når du steker fisk og/eller tilbehør til fisk: (sett ett kryss)

☐ Steker uten fett ☐ Soyaolje
☐ Smør ☐ Rapsolje
☐ Fast margarin ☐ Olivenolje
☐ Flytende margarin ☐ Solsikkeolje
☐ Annen olje (spesifiser) ____________

Kjøtt

Hvor ofte spiser du reinkjøtt?

☐ Aldri/sjelden ☐ 1 pr. mnd. ☐ 2–3 pr. mnd.
☐ 1 pr. uke ☐ 2–3 pr. uke ☐ 4+ pr. uke

Hvor ofte spiser du følgende kjøtt- og fjærkreretter? (Sett ett kryss for hver rett)

	aldri/ sjelden	1 pr. mnd.	2–3 pr. mnd	1 pr. uke	2+ pr. uke
Steik *(okse, svin, får)*	☐	☐	☐	☐	☐
Koteletter	☐	☐	☐	☐	☐
Biff	☐	☐	☐	☐	☐
Kjøttkaker, karbonader	☐	☐	☐	☐	☐
Pølser	☐	☐	☐	☐	☐
Gryterett, lapskaus	☐	☐	☐	☐	☐
Pizza med kjøtt	☐	☐	☐	☐	☐
Kylling med skinn	☐	☐	☐	☐	☐
Kylling uten skinn	☐	☐	☐	☐	☐
Bacon, flesk	☐	☐	☐	☐	☐
Andre kjøttretter	☐	☐	☐	☐	☐

Dersom du spiser følgende retter, oppgi du vanligvis spiser: (Sett ett kryss for hver linje)

Steik *(skiver)* ☐ 1 ☐ 2 ☐ 3
Koteletter*(stk.)* ☐ ½ ☐ 1 ☐ 1½
Kjøttkaker, karbonader *(stk.)* ☐ 1 ☐ 2 ☐ 3
Pølser *(stk. à 150g)* ☐ ½ ☐ 1 ☐ 1½
Gryterett, lapskaus *(dl)* ☐ 1–2 ☐ 3 ☐ 4
Pizza m/kjøtt *(stykke à 100 g)* ☐ 1 ☐ 2 ☐ 3

Hvilke sauser bruker du til kjøttretter og p
(Sett ett kryss pr. linje)

	aldri/ sjelden	1 pr. mnd.	2–3 pr. m
Brun saus	☐	☐	☐
Sjysaus	☐	☐	☐
Tomatsaus	☐	☐	☐
Saus med fløte/rømme	☐	☐	☐

Hvor mye bruker du vanligvis av disse sa

Brun saus *(dl)* ☐ ¼ ☐ ½ ☐ ¾
Sjysaus *(dl)* ☐ ¼ ☐ ½ ☐ ¾
Tomatsaus *(dl)* ☐ ¼ ☐ ½ ☐ ¾
Saus med fløte/rømme *(dl)* ☐ ¼ ☐ ½ ☐ ¾

Andre matvarer

Hvor mange egg spiser du vanligvis i løp uke?(stekte, kokte, eggerøre, omelett) (Sett ett kryss)

☐ 0 ☐ 1 ☐ 2 ☐ 3–4 ☐ 5–6 ☐

Hvor ofte spiser du iskrem? (til dessert, Kron
Sett ett kryss for hvor ofte du spiser iskrem om somme
kryss for resten av året

	aldri/ sjelden	1 pr. mnd.	2–3 pr. m
Om sommeren	☐	☐	☐
Resten av året	☐	☐	☐

Hvor mye is spiser du vanligvis pr. gang?
(Sett ett kryss)

☐ 1 dl ☐ 2 dl ☐ 3 dl ☐

Dersom du spiser følgende retter, oppgi mengden du vanligvis spiser: (Sett ett kryss for hver linje)

Steik *(skiver)*	☐ 1	☐ 2	☐ 3	☐ 4	☐ 5+
Koteletter *(stk.)*	☐ ½	☐ 1	☐ 1½	☐ 2+	
Kjøttkaker, karbonader *(stk.)*	☐ 1	☐ 2	☐ 3	☐ 4+	
Pølser *(stk. à 150g)*	☐ ½	☐ 1	☐ 1½	☐ 2+	
Gryterett, lapskaus *(dl)*	☐ 1–2	☐ 3	☐ 4	☐ 5+	
Pizza m/kjøtt *(stykke à 100 g)*	☐ 1	☐ 2	☐ 3	☐ 4+	

Hvilke sauser bruker du til kjøttretter og pastaretter?
(Sett ett kryss pr. linje)

	aldri/ sjelden	1 pr. mnd.	2–3 pr. mnd	1 pr. uke	2+ pr. uke
Brun saus	☐	☐	☐	☐	☐
Sjysaus	☐	☐	☐	☐	☐
Tomatsaus	☐	☐	☐	☐	☐
Saus med fløte/rømme	☐	☐	☐	☐	☐

Hvor mye bruker du vanligvis av disse sausene?

Brun saus *(dl)*	☐ ¼	☐ ½	☐ ¾	☐ 1	☐ 2+
Sjysaus *(dl)*	☐ ¼	☐ ½	☐ ¾	☐ 1	☐ 2+
Tomatsaus *(dl)*	☐ ¼	☐ ½	☐ ¾	☐ 1	☐ 2+
Saus med fløte/rømme *(dl)*	☐ ¼	☐ ½	☐ ¾	☐ 1	☐ 2+

Andre matvarer

Hvor mange egg spiser du vanligvis i løpet av en uke? (stekte, kokte, eggerøre, omelett) (Sett ett kryss)

☐ 0 ☐ 1 ☐ 2 ☐ 3–4 ☐ 5–6 ☐ 7+

Hvor ofte spiser du iskrem? (til dessert, Krone-is osv.)
Sett ett kryss for hvor ofte du spiser iskrem om sommeren, og ett kryss for resten av året

	aldri/ sjelden	1 pr. mnd.	2–3 pr. mnd	1 pr. uke	2+ pr. uke
Om sommeren	☐	☐	☐	☐	☐
Resten av året	☐	☐	☐	☐	☐

Hvor mye is spiser du vanligvis pr. gang?
(Sett ett kryss)

☐ 1 dl ☐ 2 dl ☐ 3 dl ☐ 4+ dl

Hvor ofte spiser du bakevarer som boller, kaker, wienerbrød eller småkaker (Sett ett kryss pr. linje)

	aldri/ sjelden	1–3 pr. mnd	1 pr. uke	2–3 pr. uke	4–6 pr. uke	1+ pr. dag
Gjærbakst (boller o.l.)	☐	☐	☐	☐	☐	☐
Wienerbrød, kringle	☐	☐	☐	☐	☐	☐
Kaker	☐	☐	☐	☐	☐	☐
Pannekaker	☐	☐	☐	☐	☐	☐
Vafler	☐	☐	☐	☐	☐	☐
Småkaker, kjeks	☐	☐	☐	☐	☐	☐
Lefser, lomper	☐	☐	☐	☐	☐	☐

Hvor ofte spiser du dessert? (Sett ett kryss pr. linje)

	aldri/ sjelden	1 pr. mnd	2–3 pr. mnd	1 pr. uke	2–3 pr. uke	4+ pr.uke
Pudding sjokolade/karamell	☐	☐	☐	☐	☐	☐
Riskrem, fromasj	☐	☐	☐	☐	☐	☐
Kompott, fruktgrøt, hermetisk frukt	☐	☐	☐	☐	☐	☐
Jordbær *(friske, frosne)*	☐	☐	☐	☐	☐	☐
Andre bær *(friske, frosne)*	☐	☐	☐	☐	☐	☐

Hvor ofte spiser du sjokolade? (Sett ett kryss)

	aldri/ sjelden	1–3 pr. mnd	1 pr. uke	2–3 pr. uke	4–6 pr. uke	1+ pr.dag
Mørk sjokolade	☐	☐	☐	☐	☐	☐
Lys sjokolade	☐	☐	☐	☐	☐	☐

Dersom du spiser sjokolade, hvor mye pleier du vanligvis å spise hver gang? Tenk deg størrelsen på en Kvikk-Lunsj sjokolade, og oppgi hvor mye du spiser i forhold til den.

☐ ¼ ☐ ½ ☐ ¾ ☐ 1 ☐ 1½ ☐ 2+

Hvor ofte spiser du snacks? (Sett ett kryss)

	aldri/ sjelden	1–3 pr. mnd	1 pr. uke	2–3 pr. uke	4–6 pr. uke	1+ pr.dag
Potetchips	☐	☐	☐	☐	☐	☐
Peanøtter	☐	☐	☐	☐	☐	☐
Andre nøtter	☐	☐	☐	☐	☐	☐
Annen snacks	☐	☐	☐	☐	☐	☐

Tran og fiskeoljekapsler

Bruker du tran (flytende)?

☐ Ja ☐ Nei

Hvis ja; hvor ofte tar du tran? Sett ett kryss for hver linje.

	aldri/ sjelden	1–3 pr. mnd	1 pr. uke	2–6 pr. uke	daglig
Om vinteren	☐	☐	☐	☐	☐
Resten av året	☐	☐	☐	☐	☐

Hvor mye tran pleier du å ta hver gang?

☐ 1 ts ☐ ½ ss ☐ 1+ss

Bruker du tranpiller/fiskeoljekapsler?

☐ Ja ☐ Nei

Hvis ja; hvor ofte tar du tranpiller/fiskeoljekapsler?
Sett ett kryss for hver linje.

	aldri/ sjelden	1–3 pr. mnd	1 pr. uke	2–6 pr. uke	daglig
Om vinteren	☐	☐	☐	☐	☐
Resten av året	☐	☐	☐	☐	☐

Hvilken type tranpiller/fiskeoljekapsler bruker du vanligvis, og hvor mange pleier du å ta hver gang?

Navn: ______________________________

Antall: ☐ 1 ☐ 2 ☐ 3+

Kosttilskudd

Bruker du kosttilskudd (vitaminer/mineraler)?
☐ Ja ☐ Nei

Hvis ja, hvor ofte bruker du kosttilskudd?
(Sett ett kryss pr. linje)

Navn på kosttilskudd	aldri/ sjelden	1–3 pr. mnd	1 pr. uke	2–6 pr. uke	daglig
______________	☐	☐	☐	☐	☐
______________	☐	☐	☐	☐	☐
______________	☐	☐	☐	☐	☐
______________	☐	☐	☐	☐	☐

Alkohol

Er du totalavholdskvinne?
☐ Ja ☐ Nei

Hvis Nei; hvor ofte og hvor mye drakk du i gjennomsnitt siste året? (Sett ett kryss for hver linje)

	aldri/ sjelden	1 pr. mnd	2–3 pr. mnd.	1 pr. uke	2–4 pr. uke	5–6 pr. uke	1 pr. dag	2+ pr. dag
Øl *(½ l.)*	☐	☐	☐	☐	☐	☐	☐	☐
Vin *(glass)*	☐	☐	☐	☐	☐	☐	☐	☐
Brennevin *(drink)*	☐	☐	☐	☐	☐	☐	☐	☐
Likør/Hetvin *(glass)*	☐	☐	☐	☐	☐	☐	☐	☐

Sosiale forhold

Hvor mange personer er det i ditt hushold?
☐ 1 ☐ 2 ☐ 3 ☐ 4 ☐ 5+

Hvor høy er bruttoinntekten i husholdet pr. år?
☐ inntil 150.000 kr.
☐ 151.000–300.000 kr.
☐ 301.000–450.000 kr
☐ 451.000–600.000 kr.
☐ 601.000–750.000 kr.
☐ 751.000–900.000 kr.
☐ over 900.000 kr.

Solvaner

Hvor mange ganger pr. år er du blitt forbrent av solen slik at du har fått svie eller blemmer med avflassing etterpå?

Årstall	Aldri	Høyst 1 g. pr. år	2–3 g. pr. år	4–5 g. pr. år	6 eller flere g. pr. år
2003–2011	☐	☐	☐	☐	☐

Hvor mange uker i gjennomsnitt pr. år har du vært på badeferie 2003–2011?

Årstall	Aldri	1 uke	2–3 uker	4–5 uker	7 uker eller mer
I syden	☐	☐	☐	☐	☐
I Norge	☐	☐	☐	☐	☐

Hvor ofte har du solt deg i solarium?

Årstall	Aldri	Sjelden	1 g. pr. mnd.	2 g. pr. mnd.	3–4 g. pr. mnd.	Oftere enn 1 g. pr. mnd.
2003–2011	☐	☐	☐	☐	☐	☐

Hvor ofte dusjer eller bader du?

	mer enn 1 g. dagl	1 g. dagl.	4–6 g. pr. uke	2–3 g. pr. uke	1 g. pr. uke	2–3 g. pr. mnd.	sjelden/ aldri
Med såpe/ shampo	☐	☐	☐	☐	☐	☐	☐
Uten såpe/ shampo	☐	☐	☐	☐	☐	☐	☐

Når bruker du krem med solfaktor? (sett evt. flere kryss):
☐ i påsken
☐ solferie i syden
☐ i Norge eller utenfor syden
☐ aldri

Hvilken solfaktor bruker du i disse periodene?

Faktor	Ingen	1–4	5–9	10–14	15–29	30+
Påsken	☐	☐	☐	☐	☐	☐
I Norge eller utenfor syden	☐	☐	☐	☐	☐	☐
Solferie i syden	☐	☐	☐	☐	☐	☐

Hvor ofte bruker du følgende hudpleiemidler?
(Sett ett kryss pr. linje)

	aldri/ sjelden	1–3 pr. mnd.	1 pr. uke	2–4 pr. uke	5–6 pr. uke	1 pr. dag	2+ pr. dag
Ansiktskrem	☐	☐	☐	☐	☐	☐	☐
Håndkrem	☐	☐	☐	☐	☐	☐	☐
Body lotion	☐	☐	☐	☐	☐	☐	☐
Parfyme	☐	☐	☐	☐	☐	☐	☐

Til slutt vil vi spørre deg om ditt samtykke til å kontakte deg på nytt pr. post. Vi vil hente adressen fra det sentrale personregister ☐ Ja ☐ Nei

Er du villig til å avgi en blodprøve? ☐ Ja ☐ Nei

Takk for at du ville delta i undersøkelsen

Institutt for samfunnsmedisin
Universitetet i Tromsø
9037 Tromsø
Telefon 77 64 48 16 / 77 64 66 38

KVINNER OG KR

Orientering om undersøk

Du samtykket i 2003 til å fylle ut e
maet tok opp en rekke forhold knyt
forhold. Formålet med undersøkels
kvinner. Resultatene vil bli publiser
søkelsen er professor Eiliv Lund.

Vi retter nå en ny forespørsel til
Begrunnelsen for å kontakte deg på
som vi vet endrer seg med alderen.

Undersøkelsen er tilrådd av Reg
din henter vi fra det sentrale person
der spørreskjemaet kun løpenumm
personvern.

Med noen års mellomrom frem
søkelsen med opplysninger fra Kre
håper vi å finne ut årsakene til at n
registrene vil bli behandlet konfide

Det er frivillig om du vil være n
at det vil få noen konsekvenser for

Vi vil be deg om å besvare det v
gitte svaralternativ dekker din situa
merknader eller tilleggsopplysning
om noen år i form av et liknende s

Vi vil senere kontakte en del av
være gratis. Noen kvinner vil også

For spørsmål om bruk av hormo
et hjelpemiddel (brosjyren skal ikk
betaler svarporto for.

Eiliv Lund
professor dr.med.

Du kan finne mer informasjon om
