# Supplementary figures and images for: Overall lifestyle changes in adulthood are associated with cancer incidence in the Norwegian Women and Cancer Study (NOWAC) – a prospective cohort study
Source: BMC Public Health. 2023 Apr 3;23:633. doi: 10.1186/s12889-023-15476-3 (PMC10069035; doi:10.1186/s12889-023-15476-3)

**Additional File 3.**  
Sample flow chart,  
Norwegian Women and  
Cancer Study (NOWAC)

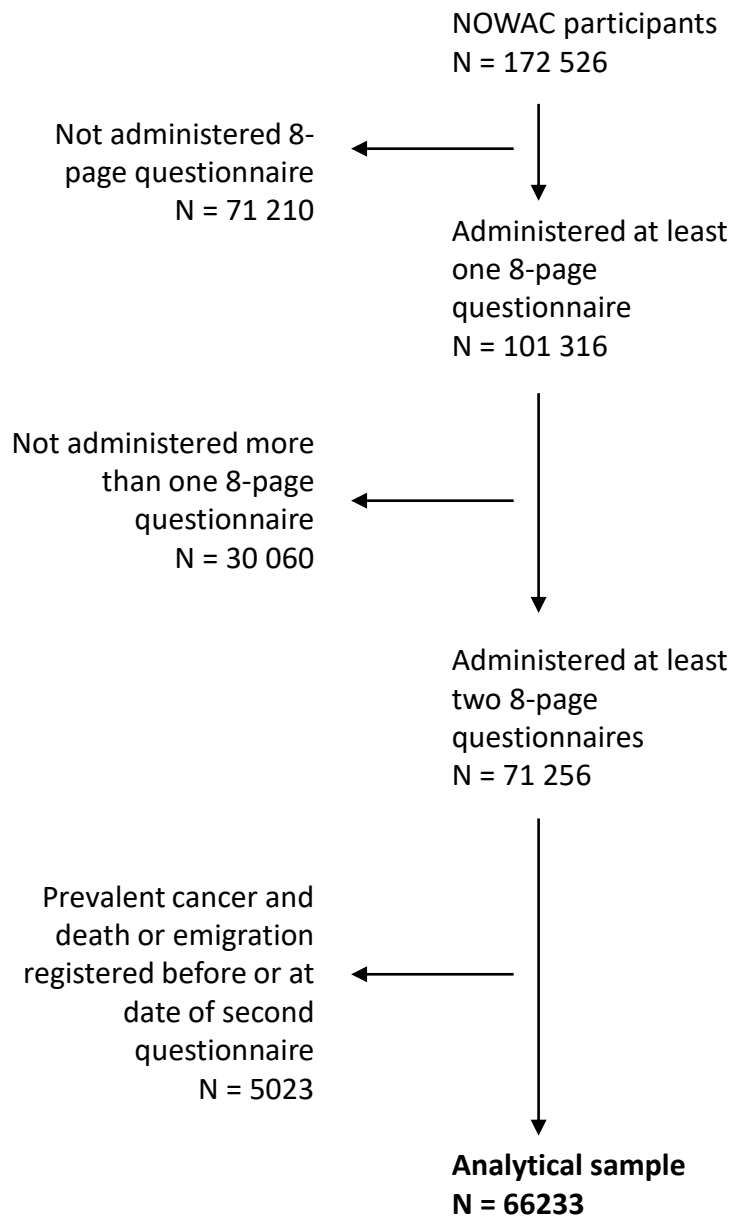

Supplement: Supplementary file 3 — Additional file 3. Sample flowchart, Norwegian Women and Cancer Study (NOWAC). [file 12889_2023_15476_MOESM3_ESM.pdf]

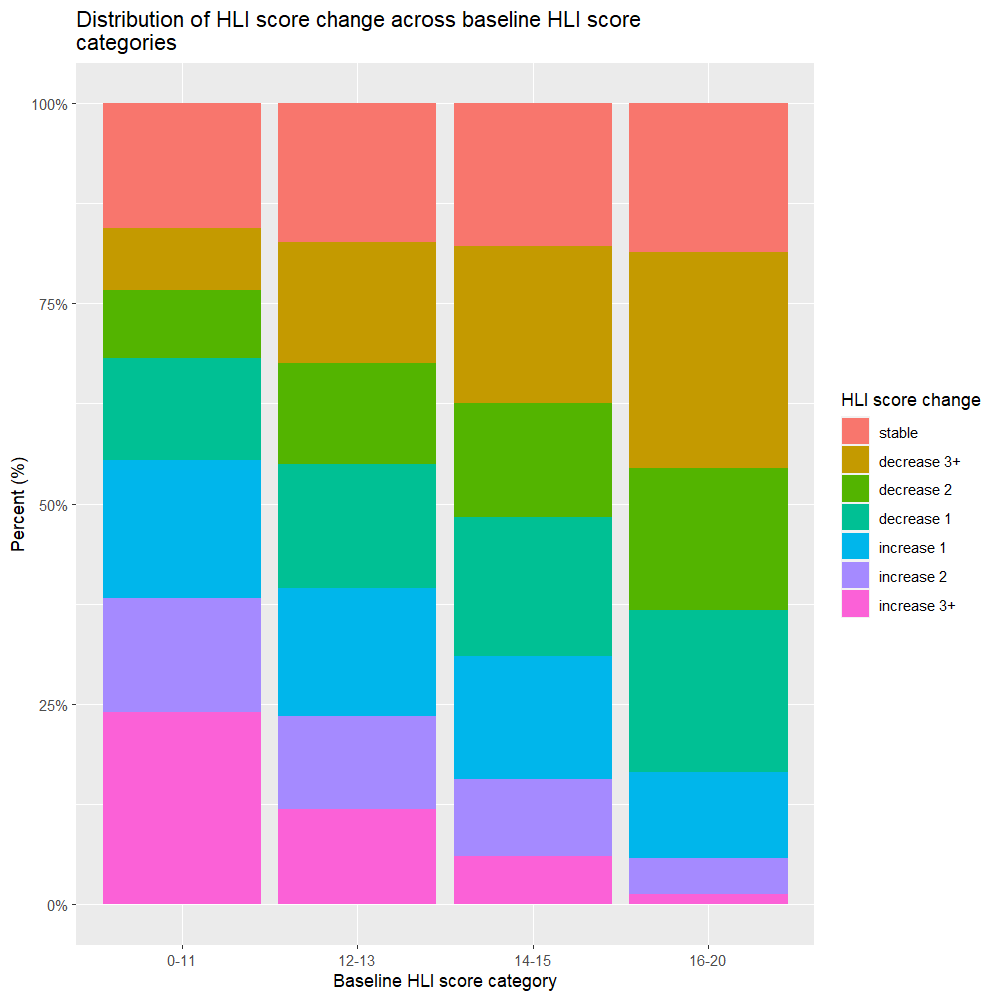

Supplement: Supplementary file 6 — Additional file 6. Distribution of HLI score change across baseline HLI score categories. [file 12889_2023_15476_MOESM6_ESM.png]

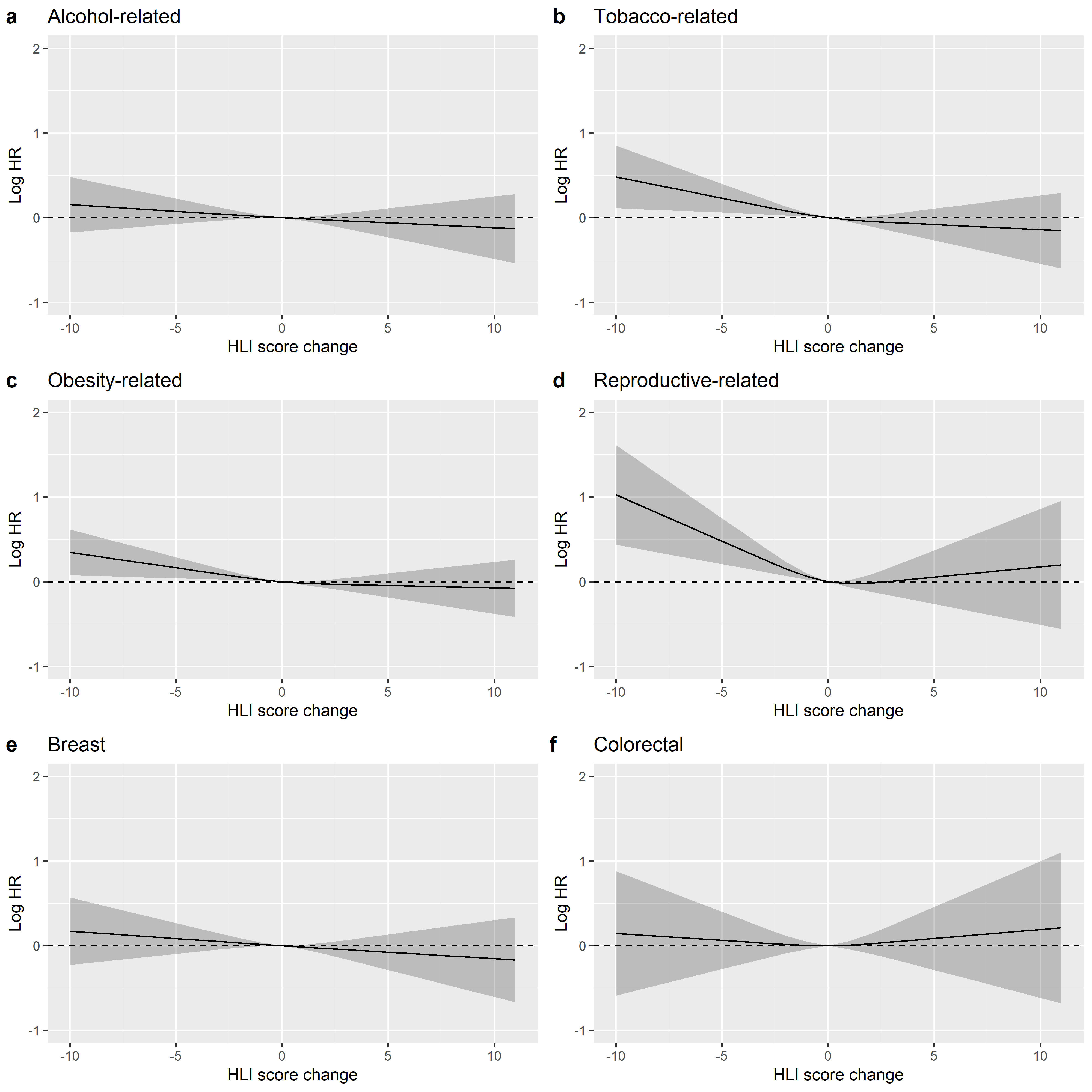

Supplement: Supplementary file 8 — Additional file 8. Associations between HLI score change modelled with restricted cubic splines and several cancer subgroupings. [file 12889_2023_15476_MOESM8_ESM.png]
