# Supplementary material for: Overall lifestyle changes in adulthood are associated with cancer incidence in the Norwegian Women and Cancer Study (NOWAC) – a prospective cohort study
Source: BMC Public Health. 2023 Apr 3;23:633. doi: 10.1186/s12889-023-15476-3 (PMC10069035; doi:10.1186/s12889-023-15476-3)
Supplement: Supplementary file 4 — Additional file 4. Description of healthy lifestyle index (HLI) construction in the Norwegian Women and Cancer Study (NOWAC). [file 12889_2023_15476_MOESM4_ESM.docx]

**Additional File 4**

**Description of healthy lifestyle index (HLI) construction in the Norwegian Women and Cancer study (NOWAC)**

Physical activity level was reported by participants on a 10-point scale ranging from very little activity to very active, where participants were asked to consider the entirety of activity at work, outside work, at home, exercise, and other forms of physical activity. Since this measure could not be categorized according to physical activity guidelines or other measures of the dose of physical activity, physical activity level was scored by quintile based on the percentile distribution at Q1 (physical activity scale 7-10 = 4, 6 = 3, 5 = 2, 4 = 1, 1-3 = 0). Body fatness was assessed through self-reported height (centimeters) and weight (kilograms) to calculate body mass index (kg/m^2^) (BMI <23 = 4, 23 to <25 = 3, 25 to <27 = 2, 27 to <30= 1, ≥30 = 0), smoking was scored considering smoking status, smoking intensity and time since cessation (never smoker = 4, former smoker >10 years since cessation = 3, former smoker ≤10 years since cessation = 2, smoker <15 cigarettes/day = 1, current smoker ≥15 cigarettes/day = 0), and alcohol (ethanol) consumption was recorded in grams/day (none = 4, >0 to <5 = 3, 5 to <10 = 2, 10 to <20 = 1, >20 = 0). A diet score ranging from 0 to 18 (healthiest) was generated, comprising six food groups: whole grains, fruit, vegetables, dairy, red meat, and processed meat. Using an analysis program developed at the Institute of Community Medicine, UiT The Arctic University of Norway, daily intake of food groups and energy were computed based on the frequencies and portions of food items reported in the FFQ according to the food composition table for Norway (1). Each food group was adjusted for energy intake, by dividing grams of intake by daily energy intake, in millijoules (MJ). The energy-adjusted food groups were categorized into quartiles and scored from 0 (lowest quartile) to 3 (highest quartile). Red and processed meat were scored in reverse order. The 18-point diet score was then divided into quintiles to produce a score ranging from 0 to 4 for inclusion in the HLI (11-18 = 4, 10 = 3, 8-9 = 2, 7 = 1, 0-6 = 0).

References

1. The Norwegian Food Composition Database 2021 [Internet]. 2021. Available from: <https://www.matvaretabellen.no/>.
