# Supplementary material for: Overall lifestyle changes in adulthood are associated with cancer incidence in the Norwegian Women and Cancer Study (NOWAC) – a prospective cohort study
Source: BMC Public Health. 2023 Apr 3;23:633. doi: 10.1186/s12889-023-15476-3 (PMC10069035; doi:10.1186/s12889-023-15476-3)
Supplement: Supplementary file 7 — Additional file 7. Associations between healthy lifestyle index score change and lifestyle-related, alcohol-related, tobacco-related, obesity-related, reproductive-related, breast, and colorectal cancer incidence in the Norwegian Women and Cancer Study (n = 44404), complete-case analysis. [file 12889_2023_15476_MOESM7_ESM.docx]

Associations between healthy lifestyle index score change and lifestyle-related, alcohol-related, tobacco-related, obesity-related, reproductive-related, breast, and colorectal cancer incidence in the Norwegian Women and Cancer Study (n=44404), complete-case analysis

|  |  | Lifestyle-related cancer incidence^a^ | Alcohol-related cancer incidence^a^ | Tobacco-related cancer incidence | Obesity-related cancer incidence^a^ | Reproductive-related cancer incidence^a^ | Breast cancer incidence^a^ | Colorectal cancer incidence |
| --- | --- | --- | --- | --- | --- | --- | --- | --- |
| Cases |  | 3926 | 2348 | 1805 | 3309 | 611 | 1567 | 468 |
| Continuous HLI score change | 1-SD (2.4 HLI points) increase | 0.94(0.92-0.97) | 0.97(0.94-1.01) | 0.94(0.90-0.98) | 0.96(0.93-0.99) | 0.91(0.85-0.98) | 0.97(0.83-1.01) | 1.00(0.93-1.09) |
| Categorical HLI score change | <= -3 | 1.18(1.06-1.32) | 1.07(0.93-1.24) | 1.27(1.08-1.50) | 1.12(0.99-1.26) | 1.31(0.99-1.72) | 1.06(0.89-1.26) | 1.25(0.91-1.73) |
|  | -2 | 1.12(1.00-1.27) | 1.16(1.00-1.34) | 1.16(0.97-1.38) | 1.12(0.98-1.27) | 1.14(0.85-1.53) | 1.14(0.95-1.36) | 1.14(0.80-1.61) |
|  | -1 | 1.03(0.92-1.14) | 1.04(0.90-1.20) | 1.07(0.91-1.27) | 0.99(0.88-1.12) | 0.96(0.72-1.28) | 1.03(0.87-1.23) | 1.10(0.79-1.53) |
|  | 0 | 1.00 (ref) |  |  |  |  |  |  |
|  | 1 | 0.98(0.87-1.09) | 0.96(0.83-1.11) | 1.09(0.93-1.29) | 0.96(0.85-1.08) | 0.98(0.74-1.30) | 0.91(0.76-1.09) | 1.19(0.86-1.65) |
|  | 2 | 0.92(0.81-1.04) | 0.98(0.83-1.15) | 0.97(0.80-1.16) | 0.95(0.83-1.08) | 0.84(0.60-1.16) | 0.96(0.79-1.16) | 1.07(0.74-1.54) |
|  | >=3 | 0.97(0.86-1.09) | 0.98(0.84-1.15) | 1.01(0.85-1.21) | 0.96(0.84-1.09) | 1.05(0.78-1.41) | 0.94(0.78-1.14) | 1.21(0.86-1.72) |
| HLI score change excluding one factor^b^ | 1-SD increase |  |  |  |  |  |  |  |
| Excluding physical activity | 1.9 | 0.96(0.93-0.99) | 0.94(0.93-1.02) | 0.96(0.91-1.01) | 0.96(0.93-1.00) | 0.94(0.86-1.02) | 0.97(0.92-1.03) | 0.99(0.90-1.09) |
| Excluding BMI | 2.2 | 0.93(0.90-0.96) | 0.97(0.93-1.01) | 0.88(0.83-0.92) | 0.97(0.94-1.01) | 0.97(0.89-1.06) | 0.97(0.92-1.03) | 0.99(0.90-1.10) |
| Excluding smoking | 2.4 | 0.95(0.92-0.98) | 0.97(0.93-1.02) | 0.98(0.94-1.04) | 0.95(0.91-0.98) | 0.87(0.79-0.94) | 0.96(0.90-1.01) | 1.02(0.92-1.13) |
| Excluding alcohol | 2.3 | 0.93(0.90-0.96) | 0.98(0.94-1.02) | 0.93(0.88-0.97) | 0.95(0.91-0.98) | 0.86(0.79-0.94) | 0.98(0.92-1.03) | 1.01(0.92-1.11) |
| Excluding diet | 1.9 | 0.93(0.90-0.96) | 0.96(0.92-1.00) | 0.93(0.89-0.98) | 0.95(0.91-0.98) | 0.92(0.84-1.00) | 0.95(0.90-1.00) | 1.01(0.91-1.11) |
| Single HLI factors^c^ | 1-unit increase (score 0-4) |  |  |  |  |  |  |  |
| Physical activity score change |  | 0.96(0.93-0.98) | 0.98(0.95-1.02) | 0.96(0.93-1.00) | 0.98(0.95-1.01) | 0.93(0.87-1.00) | 0.97(0.93-1.01) | 1.03(0.95-1.11) |
| BMI score change |  | 0.97(0.92-1.01) | 0.98(0.92-1.03) | 1.03(0.97-1.10) | 0.93(0.89-0.98) | 0.84(0.76-0.94) | 0.95(0.89-1.02) | 1.01(0.89-1.14) |
| Smoking score change |  | 0.98(0.93-1.04) | 1.00(0.92-1.09) | 0.94(0.86-1.02) | 1.01(0.95-1.09) | 1.08(0.91-1.27) | 1.02(0.93-1.13) | 1.05(0.87-1.26) |
| Alcohol score change |  | 1.00(0.95-1.05) | 0.93(0.87-0.99) | 1.02(0.95-1.10) | 0.99(0.93-1.04) | 1.18(1.03-1.34) | 0.91(0.85-0.99) | 0.99(0.85-1.14) |
| Diet score change |  | 0.99(0.97-1.02) | 1.01(0.97-1.04) | 1.00(0.96-1.03) | 1.00(0.97-1.02) | 0.98(0.92-1.04) | 1.00(0.96-1.04) | 1.01(0.94-1.08) |

Footnotes:

All models were adjusted for education (years), height (centimetres), HLI score at Q1 (continuous), and calendar year at Q2 (continuous). ^a^Models additionally adjusted for age at menarche (years), menopausal status (premenopausal/postmenopausal), breastfeeding (cumulative months 0, <=12, >12), hormone replacement therapy use (never/former/current), oral contraceptive use (never/ever), parity (0, 1-2, >2), and history of breast cancer in a first degree relative (yes/no). ^b^Baseline HLI score was adjusted by separately adjusting for HLI score at Q1 excluding the factor in question and the individual factor score at Q1. ^c^Mutually adjusted for all single factor HLI score changes and single factor HLI scores at Q1.

Alcohol-related cancers including sites: upper aerodigestive [C01-C10], pharynx [C11-C14], esophagus [C15], colorectum [C18-C20], liver [C22-C24], larynx [C32], breast [C50],

Tobacco-related cancers including sites: upper aerodigestive [C01-C10], pharynx [C11-C14], esophagus [C15], stomach [C16], colorectum [C18-C20], liver [C22-C24], pancreas [C25], accessory sinus [C31], larynx [C32], trachea [C33], lung [C34], breast [C50], cervix [C53], ovarian [C56], kidney [C64-C66], bladder [C67], acute myeloid leukemia [C92]

Obesity-related cancers including sites: esophagus [C15], stomach [C16], colorectum [C18-C20], liver [C22-C24], pancreas [C25], breast [C50], uterine [C54-C55], ovarian [C56], kidney [C64-C66], thyroid [C73], multiple myeloma [C90],

Reproductive-related cancers including sites: vulva [C51] vagina [C52], cervix [C53], uterine [C54-C55], ovarian [C56], other female genital organs [C57-C58],
